# Framework for X-ray mirror surface shape fitting

**DOI:** 10.1107/S1600577525011282

**Published:** 2026-01-22

**Authors:** Lei Huang, Ruochen Xu, Tianyi Wang, Jumpei Yamada, Joseph Dvorak, Corey Austin, Albert Van Eeckhout, Josep Nicolàs Roman, Kenneth Goldberg, Mourad Idir

**Affiliations:** a National Synchrotron Light Source II Brookhaven National Laboratory PO Box 5000 Upton NY11973 USA; b Ward Melville High School, 380 Old Town Road, East Setauket, NY11733, USA; c Research Center for Precision Engineering, Graduate School of Engineering Osaka University Suita Osaka565-0871 Japan; d ALBA Synchrotron Light Source Carrer de la Llum 2-26 08290Cerdanyola del Vallès Spain; e Advanced Light Source Lawrence Berkeley National Laboratory 1 Cyclotron Rd Berkeley CA94720 USA; Tohoku University, Japan

**Keywords:** X-ray mirror, mirror characterization, surface shape fitting, least-squares optimization

## Abstract

For accurate characterization of grazing-incidence X-ray mirrors, we present a comprehensive framework to fit measured surface shapes (either slope or height) of X-ray mirrors used in synchrotron radiation and free-electron laser facilities. We summarize the closed-form expressions of some typical surface shapes of X-ray mirrors including elliptic cylinders, hyperbolic cylinders, ellipsoids, hyperboloids, and diaboloids. This framework is composed of four layers: definition of standard shapes with closed-form expressions, generation of theoretical surface with pose parameters (six degrees of freedom defining an object’s position and orientation relative to a coordinate system), parameter optimization with the ability to select which parameters are fit and which are held constant, and the development of user-friendly fitting function wrappers for particular fitting tasks. A few practical fitting examples are demonstrated to verify the effectiveness of the proposed fitting framework. We discuss the physical meanings of the fitting parameters, and provide several examples using the elliptic cylinder and ellipsoid shapes to highlight some features of the framework. Moreover, we provide the presented framework as open-source codes (MATLAB and Python codes available at https://github.com/nsls2omf/xmf) to the community to encourage academic collaboration and further improvements.

## Abstract 2

The closed-form expressions for elliptic cylinders, hyperbolic cylinders, ellipsoids, hyperboloids, and diaboloids used for X-ray mirror surface shapes are summarized. Additionally, a four-layer framework to fit the measured surface shape data in slope or height for convenient X-ray mirror surface characterization is proposed.

## 1. Introduction to surface shape fitting for X-ray mirror characterization

X-ray technology plays a crucial role in scientific research and discovery at synchrotron radiation (SR) and free-electron laser (FEL) facilities (Jacobsen & Kirz, 1998 ▸; Mizutani & Suzuki, 2012 ▸; Asakura *et al.*, 2020 ▸; Yamada *et al.*, 2024 ▸). These user facilities generate highly intense X-ray beams that are used for a wide range of scientific experiments, including materials science, biology, chemistry, and other fields (Wood, 2018 ▸; Asakura *et al.*, 2020 ▸). One of the key optical components in these facilities is the X-ray mirror, which serves as a reflective optic to precisely focus and direct the beam to the endstation. The surface shape accuracy of the X-ray mirror is critical, as it directly affects the beam quality in terms of size, flux, tails, and background. Any deviations or imperfections on the mirror surface can introduce wavefront aberrations, compromising the experimental results.

It is essential to perform thorough characterization to ensure that the mirror shape meets specifications prior to their installation in beamlines. Characterization involves a series of high-precision measurements in slope or height and data analyses to assess the surface shape deviation from the target shape (Assoufid *et al.*, 2005 ▸; Yashchuk *et al.*, 2010 ▸; Qian & Idir, 2016 ▸; Vivo *et al.*, 2016 ▸; Goldberg & Yashchuk, 2016 ▸; Yashchuk *et al.*, 2019 ▸; Nakamori & Kanaoka, 2020 ▸; da Silva *et al.*, 2023 ▸; Nakamori *et al.*, 2025 ▸). One of the critical steps in this process is surface shape fitting, which precisely aligns the measured one-dimensional (1D) profile or two-dimensional (2D) surface with the desired shape. This step is crucial for identifying and evaluating deviations or imperfections. Monitoring of the mirror surface during fabrication guides the manufacturing process toward meeting the required specifications (Wang *et al.*, 2023 ▸). Proper mirror characterization with surface shape fitting helps achieve the target shape and provides a strong foundation for minimizing beam aberrations, enhancing the overall performance of X-ray focusing and imaging systems. Such measurement data and related characterization are important for beamline simulations as well to predict the performance of up-coming new beamlines or instruments.

**Figure 1:**
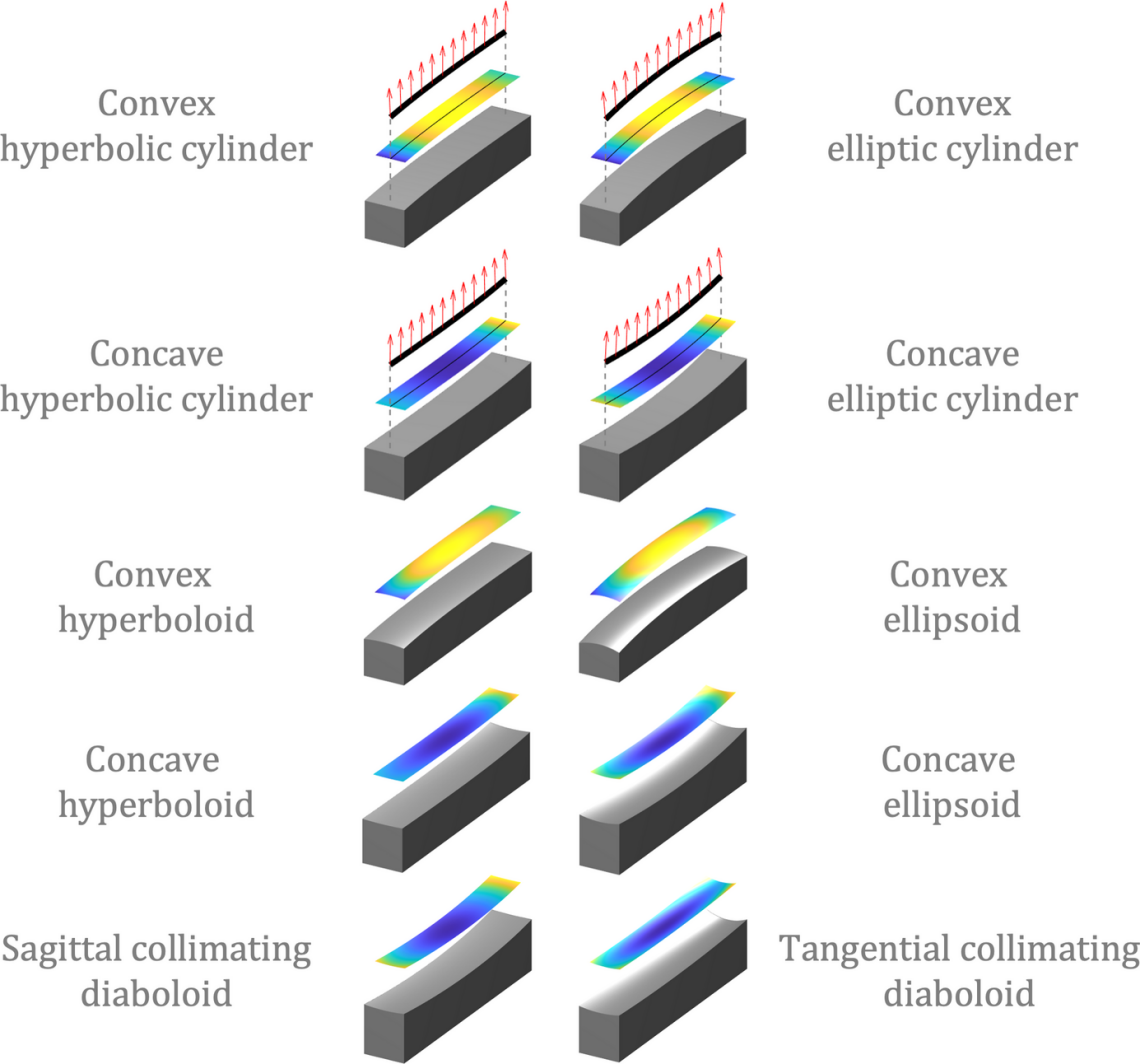
Characterization of 1D or 2D metrology data of convex and concave grazing-incidence X-ray mirrors is important for quality control in mirror fabrication and acceptance inspection.

The surface shape fitting of X-ray mirrors requires accurate mathematical expressions describing these grazing-incidence optical surfaces, as shown in Fig. 1 ▸, including but not limited to elliptic cylinders, hyperbolic cylinders, ellipsoids, hyperboloids, and diaboloids. The physical meaning of parameters in these expressions is linked to the beamline application, such as the source distance, focus distance, and the grazing-incidence angle, which are critical for achieving the most satisfactory beam size and quality. These mathematical expressions are essential for accurately fitting the surface shapes of X-ray mirrors to ensure the correct performance in focusing and directing X-ray beams. Thanks to the previous research work in the literature (McKinney *et al.*, 2011 ▸; Yashchuk *et al.*, 2021 ▸; Goldberg, 2022*a* ▸; Goldberg, 2022*b* ▸; Goldberg & Sanchez del Rio, 2023 ▸; Sanchez del Rio & Goldberg, 2024 ▸; Dvorak *et al.*, 2025 ▸), these mathematical expressions are mostly available. The sagittal collimating diaboloid is a newly derived surface shape that has not been previously published. By using these expressions, we can model the surface shapes and characterize deviations or imperfections during the fabrication and inspection processes.

While several beamline-specific surface fitting tools exist and custom mirror characterization codes are developed for particular facilities, these solutions are often optimized for a narrow range of mirror geometries or experimental conditions. They are not always openly available, and may require specialized user expertise. Our proposed framework fills this gap by providing a universal, extensible, and fully open-source approach, applicable to both 1D and 2D mirror geometries. In addition, the closed-form surface definitions ensure numerical stability and reproducibility across facilities.

In this work, we summarize the aforementioned geometric shapes used for grazing-incidence X-ray mirrors and describe a framework to fit the surface measurement in slope and height to the theoretical shapes. We will discuss the mathematical expressions for these shapes and the methods used to fit the measured surface profiles to these theoretical models. The proposed four-layer framework is user-friendly, easy to maintain, and highly adaptable for future expansion.

## 2. Framework for the fitting of X-ray mirror surface shapes

This section describes the framework (Xu *et al.*, 2025 ▸) used to fit mirror surface shape data measured with different metrology instruments. The measurement data can be tangential slope or height data from a 1D angular scanning deflectometric instrument like the Long Trace Profiler (LTP) (Takacs *et al.*, 1987 ▸; Qian *et al.*, 1995 ▸), the Nanometre Optical component Measuring machine (NOM) (Siewert *et al.*, 2004 ▸), or the Nano-accuracy Surface Profiler (NSP) (Qian & Idir, 2016 ▸; Huang *et al.*, 2020*a* ▸; Huang *et al.*, 2023 ▸) or 2D height maps from stitching interferometry (Mimura *et al.*, 2005 ▸; Yumoto *et al.*, 2008 ▸; Kimura *et al.*, 2010 ▸; Yumoto *et al.*, 2010 ▸; Vivo *et al.*, 2016 ▸; Huang *et al.*, 2020*b* ▸) or a 3D coordinate measuring machine (Handa *et al.*, 2024 ▸; Kume *et al.*, 2024 ▸).

**Figure 2:**
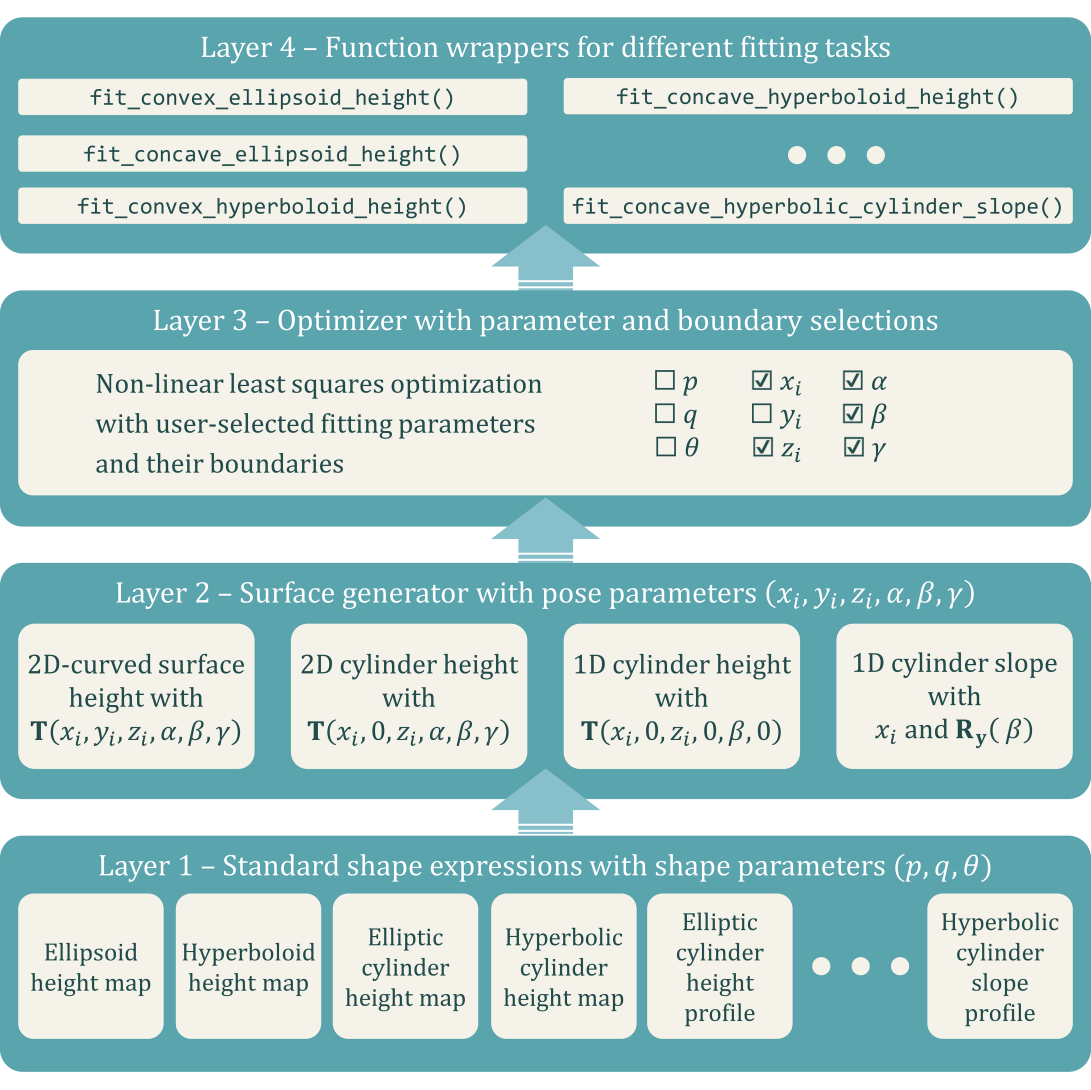
Framework from the standard shape expression to the parameter-selective shape fitting.

To handle these diverse data types, as illustrated in Fig. 2 ▸, the framework is organized into four layers from bottom to top as follows:

(i) The first (bottom) layer handles standard surface shapes defined by mathematical expressions, without translations or rotations.

(ii) The second layer is a shape generator that accounts for the mirror’s pose in the metrology instrument relative to the standard mirror coordinate system. The pose parameters include all six degrees of freedom: the chief ray intersection (three translations) and three rotation angles. In some degenerate cases, such as 1D curved shapes, certain pose parameters may not be applicable.

(iii) The third layer is a selective optimizer which minimizes the residuals between the measurements and the generated shapes in a least squares sense by adjusting a pre-selected combination of parameters.

(iv) The fourth (top) layer is a function wrapper designed for different fitting tasks. It provides user-friendly functions that allow users to conveniently fit measurement data to specific surface shapes—for example, fitting commonly used Kirkpatrick–Baez (KB) mirrors with a concave elliptic cylinder shape.

With these four layers, the framework enables flexible fitting of metrology data using user-selected parameters to optimize. Moreover, future extensions are simplified: new surface shapes can be added by defining their standard expressions in the bottom layer and wrapping them with specific functions in the top layer for convenient use. The middle layers can be reused across different but similar mirror shapes. The following subsections provide a more detailed description of each layer.

### 2.1. Standard shape expressions

The standard shape expressions of the grazing-incidence X-ray mirrors are defined in the standard mirror coordinate system (*x*_*s*_, *y*_*s*_, *z*_*s*_) with the chief ray intersection point at the origin of the mirror coordinates and no rotations applied. The standard shapes considered in this work include ellipsoids, hyperboloids, elliptic cylinders, hyperbolic cylinders, sagittal collimating diaboloids, and tangential collimating diaboloids. These shapes are described by specific mathematical equations derived in Sections S1 and S2 of the supporting information.

We summarize the mathematical expressions of standard shapes in Table 1 ▸, in which we have two conventions of expressions to describe the standard shapes:

(i) One convention uses the absolute values of the object distance |*p*| and the image distance |*q*|, along with the convex or concave property of the mirror, as described in Section S1.1 of the supporting information.

(ii) The other one, described in Section S1.2 of the supporting information, uses the object distance *p* and the image distance *q*, with signs defined according to whether the object and image are real or virtual, following the conventions of geometrical optics.

Once the standard shape *z*_*s*_(*x*_*s*_, *y*_*s*_) is defined, the next step is to generate the mirror surface considering the chief ray intersection (translations) and rotation angles.

### 2.2. Surface generation with the chief ray intersection and rotation angles

To generate the height or slope of the mirror surface for data fitting, the mirror’s pose must be taken into account through appropriate pose parameters. They include the chief ray intersection (*x*_*i*_, *y*_*i*_, *z*_*i*_) and rotation angles (α, β, γ), which are important for accurately modeling the mirror surface from actual metrology data.

(i) The chief ray intersection (*x*_*i*_, *y*_*i*_, *z*_*i*_) is the point where the chief ray of the X-ray beam intersects the mirror surface. It gives the translation from the standard mirror coordinates to the metrology coordinates.

(ii) The roll, pitch, and yaw angles—denoted by α, β, and γ, respectively—represent rotations following the right-hand rule around the tangential axis (*x*-axis in our definition), sagittal axis (*y*-axis in our definition), and the *z*-axis.

**Figure 3:**
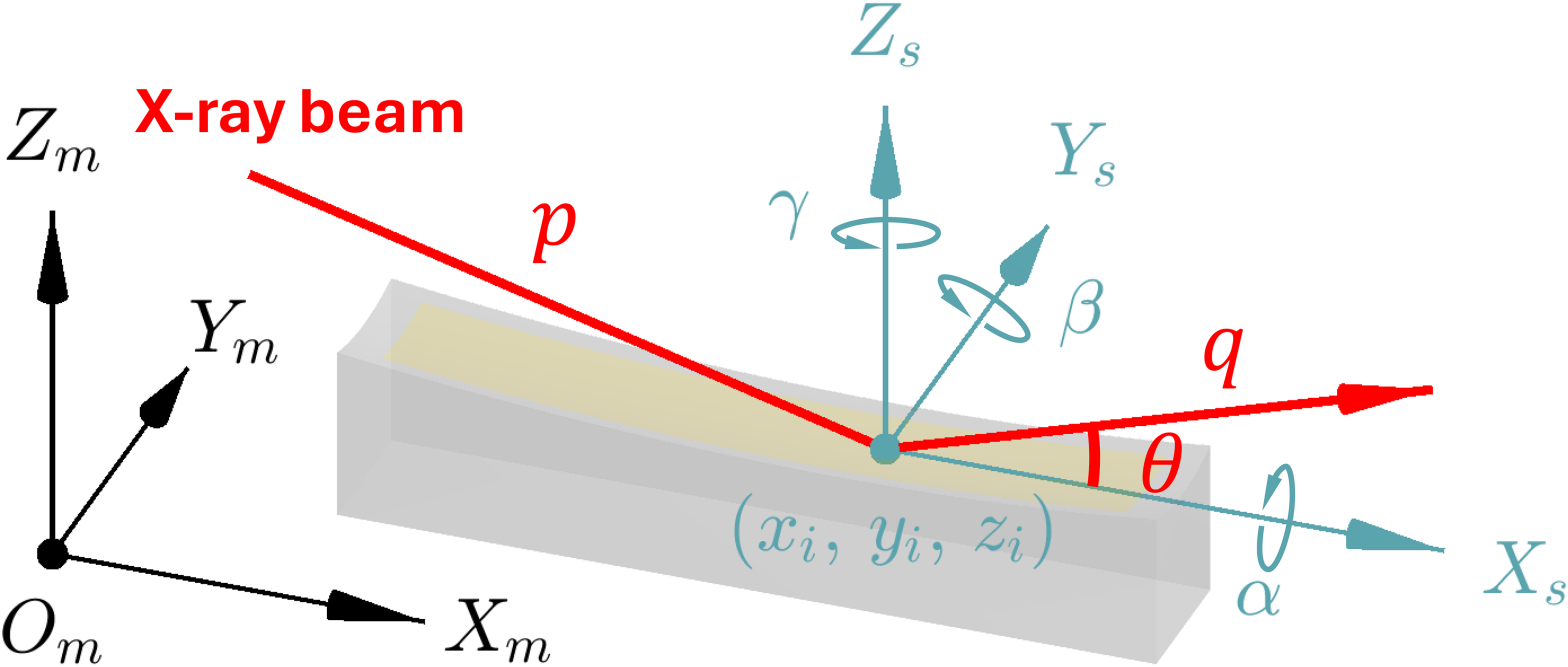
Illustration of transformation from the standard mirror coordinate system to the metrology coordinate system with the rotation angles α, β, and γ and translation vector (*x*_*i*_, *y*_*i*_, *z*_*i*_).

**Figure 4:**
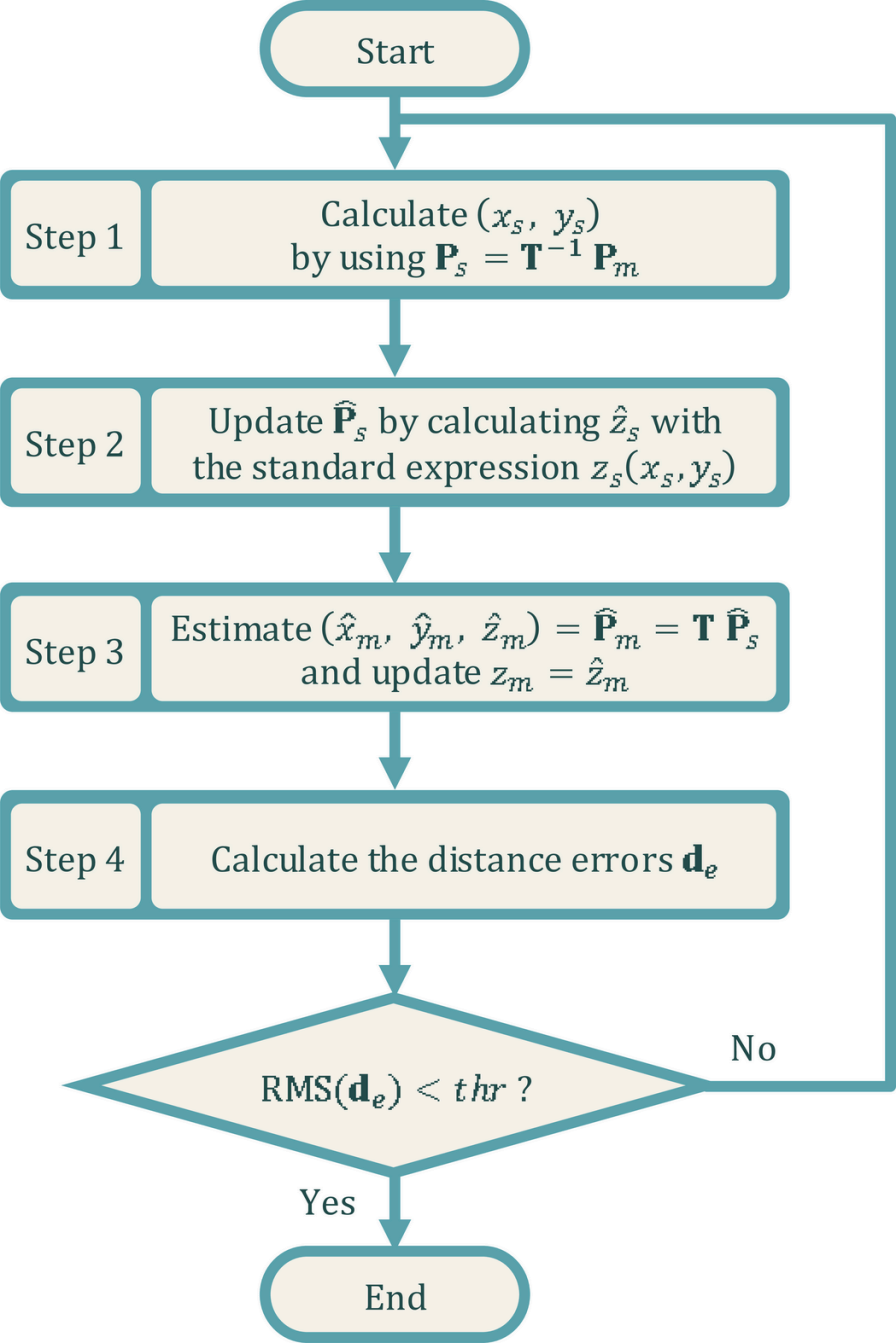
The theoretical surface shape in the measurement coordinates is generated with an iterative process.

As shown in Fig. 3 ▸, they contain all six degrees of freedom to transform an arbitrary point *P* on the mirror surface from (*x*_*s*_, *y*_*s*_, *z*_*s*_) in the standard mirror coordinates (*X*_*s*_, *Y*_*s*_, *Z*_*s*_) to (*x*_*m*_, *y*_*m*_, *z*_*m*_) in the metrology coordinates (*X*_*m*_, *Y*_*m*_, *Z*_*m*_) defined by the metrology instrument, not necessarily with the origin *O*_*m*_ at the mirror surface center. The transformation from the standard mirror coordinates to the metrology coordinates can be described as 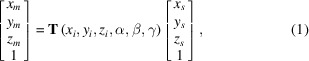
where the transformation matrix **T**(*x*_*i*_, *y*_*i*_, *z*_*i*_, α, β, γ) to transform the standard coordinates to the metrology coordinates can be expressed as 
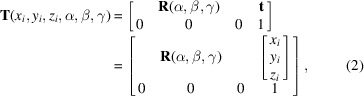
where the translation vector **t** = 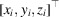 and the rotation matrix **R**(α, β, γ) is a combination of three rotation matrices along *x*, *y*, and *z* axes in sequence, 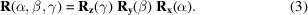
The rotation matrices **R**_*x*_(α), **R**_*y*_(β), and **R**_*z*_(γ), representing rotations along the *x*, *y*, and *z* axes, are defined as 
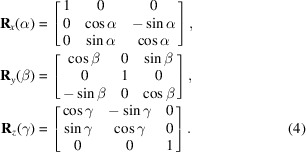
To generate the shape in the measurement data grid, one can use the known measurement location (*x*_*m*_, *y*_*m*_) in the metrology coordinate system to determine the unknown location (*x*_*s*_, *y*_*s*_) in the standard mirror coordinate system and generate the surface shape *z*_*m*_ in the metrology coordinate system through the following steps as shown in Fig. 4 ▸.

**Step 1**: Use the inverse transform **P**_*s*_ = **T**^−1^**P**_*m*_ to calculate (*x*_*s*_, *y*_*s*_).

(i) For shape generation, if *z*_*m*_ is not known, we can simply use **P**_*m*_ = 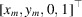 as the initial guess, since the height variation is small for X-ray mirrors.

(ii) For shape fitting, *z*_*m*_ is known from the metrology data, so **P**_*m*_ = 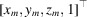 serves as a better initial value.

**Step 2**: The resultant (*x*_*s*_, *y*_*s*_) can be used to calculate the height value 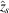 from the standard shape expression *z*_*s*_(*x*_*s*_, *y*_*s*_) in Table 1 ▸.

**Step 3**: The updated 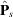 = 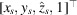 is used to estimate 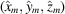 with 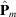 = 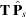. Because the *z*_*m*_ used in Step 1 is not exactly the height distribution by a forward calculation from standard mirror coordinates to the metrology coordinates, the estimated lateral coordinates 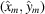 could slightly differ from the actual measurement location (*x*_*m*_, *y*_*m*_). It is possible to minimize these discrepancies with iterations with the updated *z*_*m*_ = 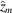.

**Step 4**: The estimated 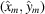 is used for comparison with (*x*_*m*_, *y*_*m*_) to evaluate the distance error *d*_*e*_ = 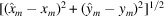 for the location of each measurement point. For all measurement points, the distance errors compose a vector **d**_*e*_.

Repeat the above steps until the root mean square (RMS) value of distance errors **d**_*e*_ is smaller than a preset threshold (*e.g.* thr = 1 × 10^−9^ m). The updated *z*_*m*_ in Step 3 is the generated height result.

To generate 2D curved mirror surfaces (like ellipsoid, hyperboloid, or diaboloid), the full parameters in **T**(*x*_*i*_, *y*_*i*_, *z*_*i*_, α, β, γ) will be used in Step 1 and Step 3 in the iteration. The generated surface is calculated as 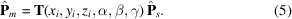
To generate 2D cylindrical mirror surfaces, *y*_*i*_ in **T** is set to 0. In iterations, the generated surface is calculated as 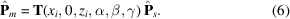
To generate 1D cylindrical height profiles, *y*_*i*_, α and γ in **T** are set to 0. In iterations, the generated surface is calculated as 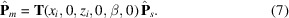
The tangential slope of a 1D elliptic or hyperbolic cylindrical mirror can be generated as 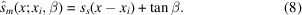
As a summary, different cases of surface generation are listed in Table 2 ▸.

The generated mirror surface serves as the theoretical target for fitting the measured data in the next step.

### 2.3. Least squares optimization with a selection of fitting parameters

The final step is to fit the generated data to the measured surface data. This step is completed by using optimization techniques that minimize the difference between the measured data and the generated data. The fitting process involves adjusting the parameters in the mathematical equations to achieve the best match with the measured data. The results include a set of fitted parameters that describe the mirror surface shape with shape parameters, (*p*, *q*, θ), and the mirror pose with pose parameters of chief ray intersection 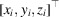 and rotation angles (α, β, γ).

Since the mirror shape expressions are nonlinear, we use a nonlinear least squares method to optimize the parameters. Therefore, the initial values are needed to start the optimization process. The initial values of the shape parameters and the chief ray intersection 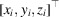 are usually set to be the values from the design. In high-precision X-ray mirror metrology, the initial values of the rotation angles (α, β, γ) are usually set to be 0 after the mirror pre-alignment in the dedicated metrology instrument.

During the optimization process, we can choose which shape parameters (*p*, *q*, θ) and pose parameters (*x*_*i*_, *y*_*i*_, *z*_*i*_, α β, γ) to include in the fitting process. This flexibility allows for tailored metrology data analysis based on specific fitting requirements. Depending on the intended application of the mirror, tolerances for certain parameters can be adjusted to accommodate acceptable residual errors, ensuring the fitting process aligns with practical performance needs, 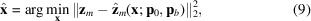
where 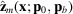 represents the shape generation described in Section 2.2. The initial parameter vector 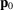 = 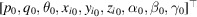 contains initial values for all parameters, with fixed parameters retaining their initial values. **p**_*b*_ can be a Boolean vector indicating which parameters are subject to optimization or a tolerance boundary to constrain the range of the parameters. **x** is the vector of **p**_*b*_-selected parameters being optimized, and 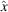 represents the final optimized parameter set. For example, **p**_*b*_ can be set as [False, False, True, True, False, True, True, True, True]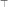 or [0, 0, Inf, Inf, 0, Inf, Inf, Inf, Inf]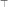 to fix *p*, *q*, and *y*_*i*_ and optimize the rest of the parameters as **x** = 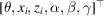. As a result, the optimized 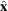 = 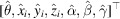 with *p* = *p*_0_ and *q* = *q*_0_ as fixed parameters in this example.

### 2.4. Function wrapper for different surface shape fitting tasks

To support specific fitting tasks efficiently, we establish convenient functions in the top layer by wrapping lower-layer components to automatically use the appropriate surface generator and standard shape. In this way, when fitting measurement data to a target shape, we need only consider the top-layer functions which provide a user-friendly interface to use the proposed fitting framework.

## 3. Demonstration of the mirror surface fitting

After describing the principle and method, we would like to demonstrate some fitting examples to show the effectiveness of the proposed X-ray mirror fitting framework. Table 3 ▸ lists fitting scenarios of some typical shapes of X-ray mirrors from actual measurements to show the practical usage of the proposed framework.

The first example is 1D slope data of a concave elliptic cylinder measured by using NSP (Qian & Idir, 2016 ▸). This 200 mm long concave elliptic cylinder is part of a KB mirror pair, which is widely used as a typical X-ray focusing optic at SR and FEL beamlines.

**Figure 5:**
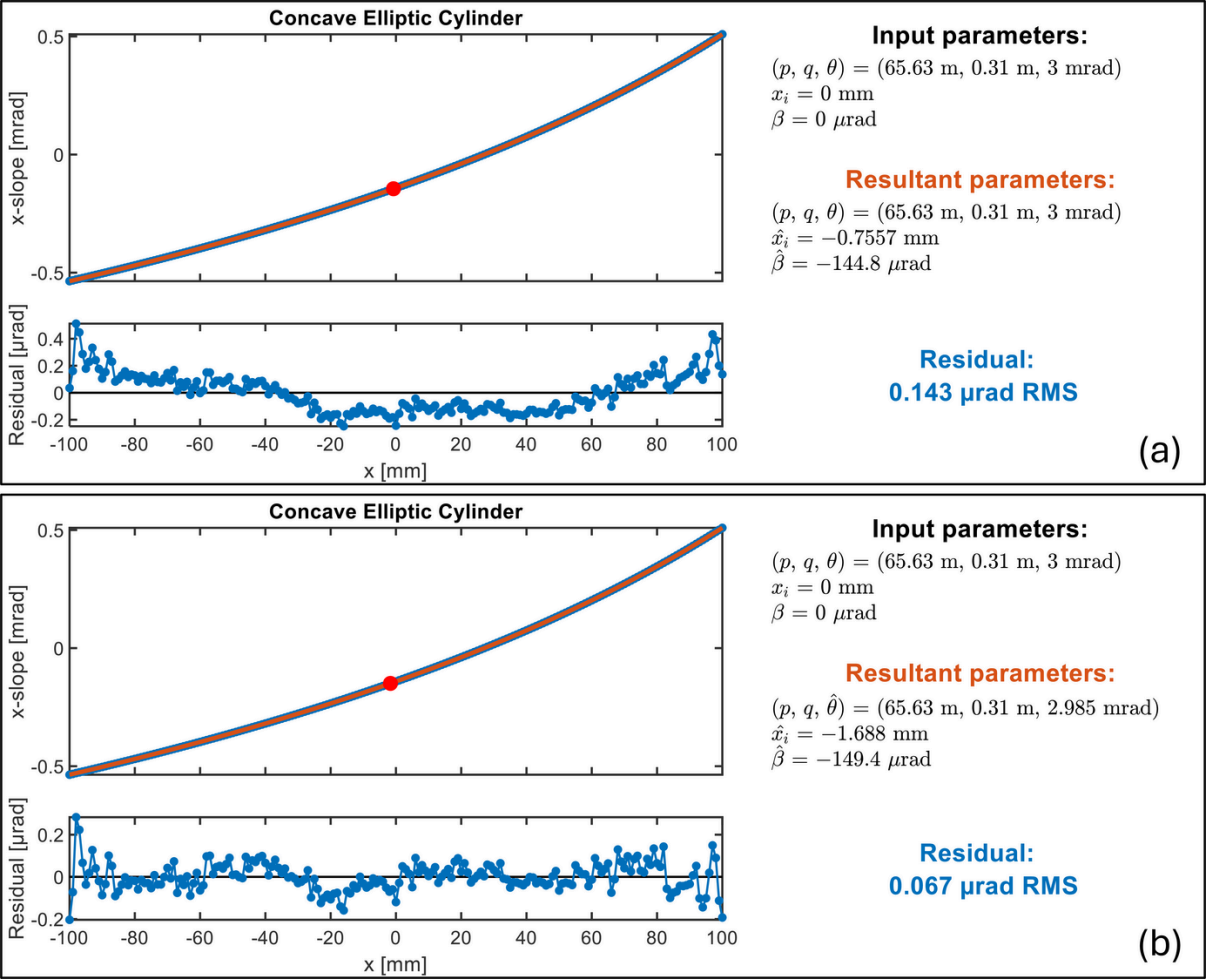
Fitting example with concave elliptic cylinder slope data. The red dot indicates the location of the chief ray intersection from the fitting. (*a*) Fitting results with fixed *p*, *q*, and θ, and optimized *x*_*i*_ and β. (*b*) Fitting results by including θ in the optimization to further minimize coma-like residuals (a second-order curve in slope).

We demonstrate two different parameter selections when fitting the 1D slope data. Parameter selection A only optimizes *x*_*i*_ and β. The chief ray intersection *x*_*i*_ is highlighted with a red dot in this 1D coordinate as shown in Fig. 5 ▸(*a*). As a result of fitting the exact target slope, the residual slope is mainly a second order curve. The other selection (selection B) chooses θ, *x*_*i*_, and β as parameters to optimize.

As shown in Fig. 5 ▸(*b*), this result gives the minimized residual slope when this mirror is used at a different grazing angle 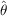 = 2.985 mrad. For some focusing applications, a small angle adjustment (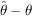 = −15 µrad in this example) is acceptable, as the vertical displacement of the focus is only about 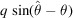 = −4.65 µm in this case.

**Figure 6:**
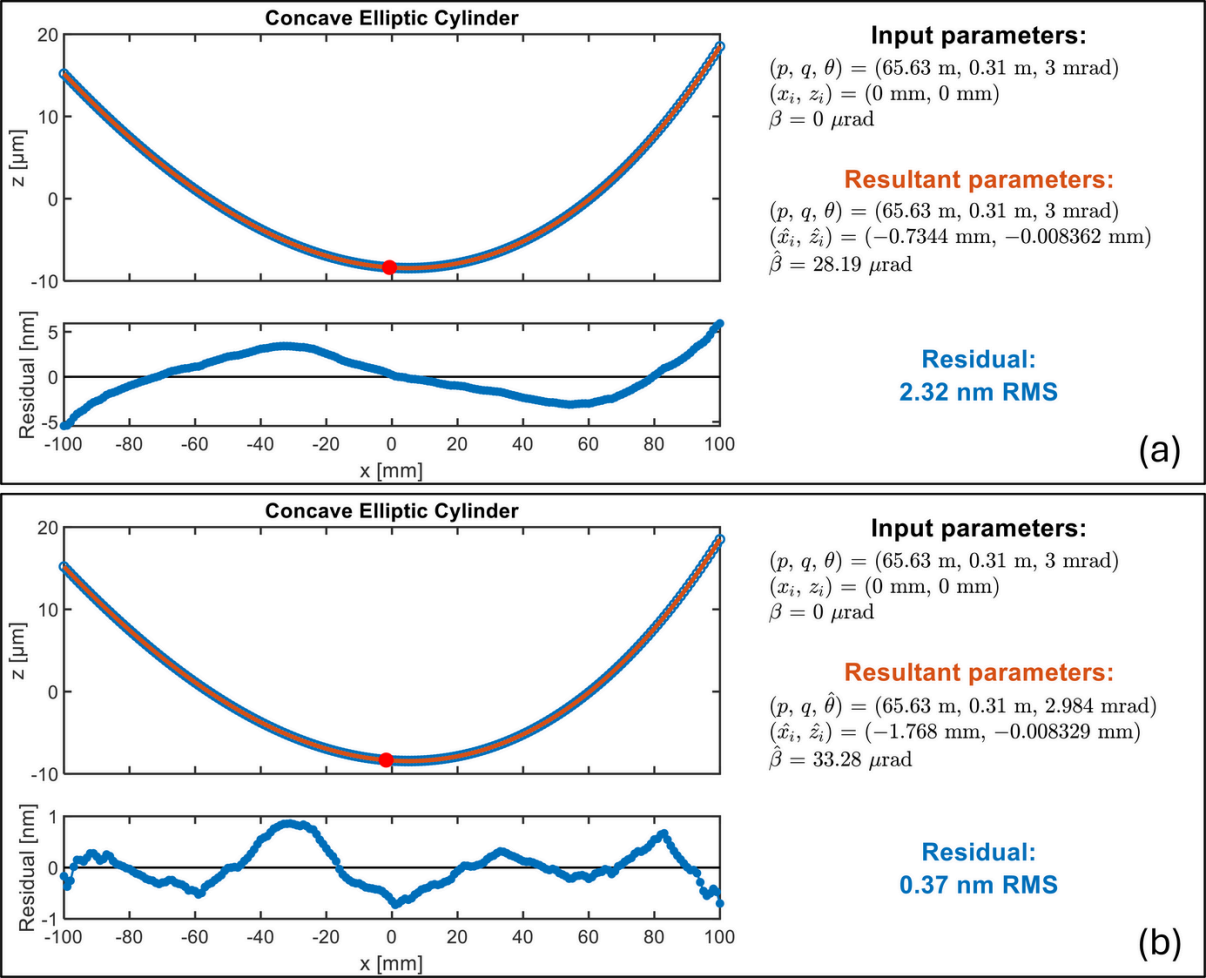
Fitting example with concave elliptic cylinder height data. The red dot indicates the location of chief ray intersection from the fitting. (*a*) Fitting results with fixed *p*, *q*, and θ, and optimized *x*_*i*_ and β. (*b*) Fitting results by including θ in the optimization to further minimize coma-like residuals.

After integration of the 1D slope data, we get 1D height data of this concave elliptic cylinder as the second fitting example. Similarly, we demonstrate two parameter selections. The first selection (selection A) optimizes *x*_*i*_, *z*_*i*_, and β to fit the height data to the exact target. As shown in Fig. 6 ▸(*a*), the chief ray intersection *x*_*i*_ is highlighted with a red dot and the residual height is a coma-like curve. The second parameter selection (selection B) includes θ, *x*_*i*_, *z*_*i*_, and β. The fitting residual is further minimized by adjusting the grazing angle to a different value 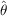 = 2.984 mrad as shown in Fig. 6 ▸(*b*).

**Figure 7:**
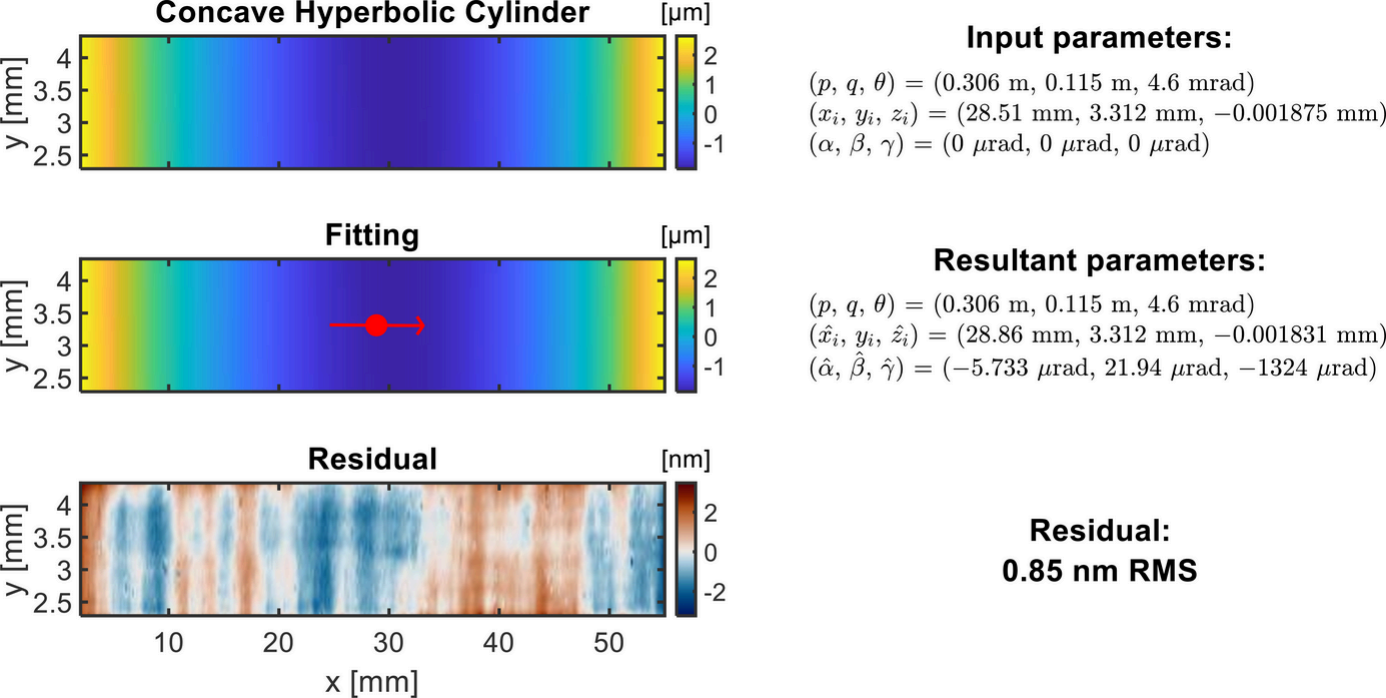
Fitting example with concave hyperbolic cylinder height data. The red dot indicates the location of the chief ray intersection from the fitting. The fitting is carried out with fixed *p*, *q*, and θ, and optimized *x*_*i*_, *z*_*i*_, α, β, and γ.

The third example is the fitting of a 2D concave hyperbolic cylinder height map, which is measured by using a coherence scanning interferometer with the micro-stitching principle (Huang *et al.*, 2024 ▸). Since this cylindrical mirror surface is almost flat in the sagittal direction, *y*_*i*_ is not selected for optimization. The other two coordinates of the chief ray intersection (*x*_*i*_, *z*_*i*_) and rotation angles (α, β, γ) are optimized to fit the stitched height surface. The optimized chief ray intersection location in 2D map (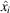, 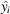) is highlighted with a red dot, and the residual height map is shown in Fig. 7 ▸.

**Figure 8:**
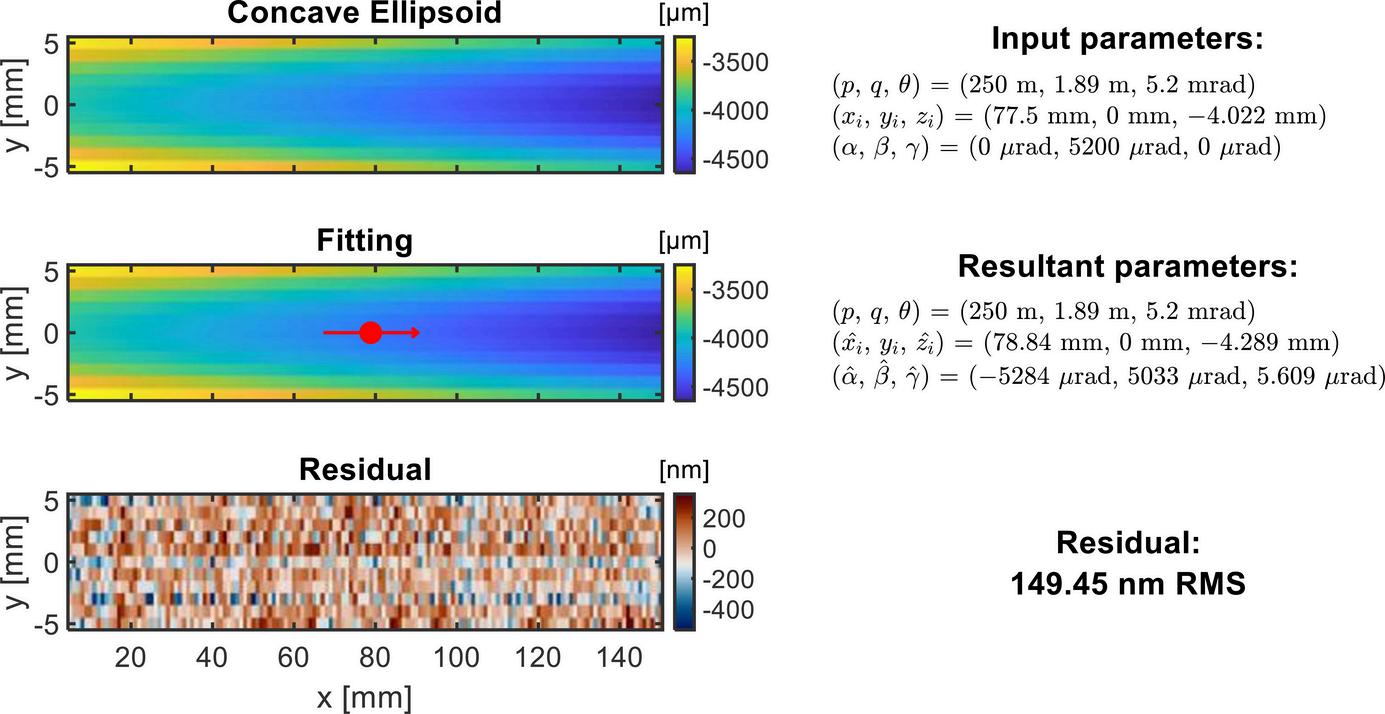
Fitting example with concave ellipsoid height data. The red dot indicates the location of the chief ray intersection from the fitting. The fitting is carried out with fixed *p*, *q*, θ, and *y*_*i*_, and optimized *x*_*i*_, *z*_*i*_, α, β, and γ.

The fourth example is the fitting of a 2D concave ellipsoidal shape. *y*_*i*_ is fixed as the mean value of the *y*-coordinates (*y*_*i*_ = 0 in this case). The other two coordinates of the chief ray intersection (*x*_*i*_, *z*_*i*_) and rotation angles (α, β, γ) are optimized to fit the measured height surface as shown in Fig. 8 ▸. The residual height shows a distribution of almost random noise intrinsic to the metrology data.

**Figure 9:**
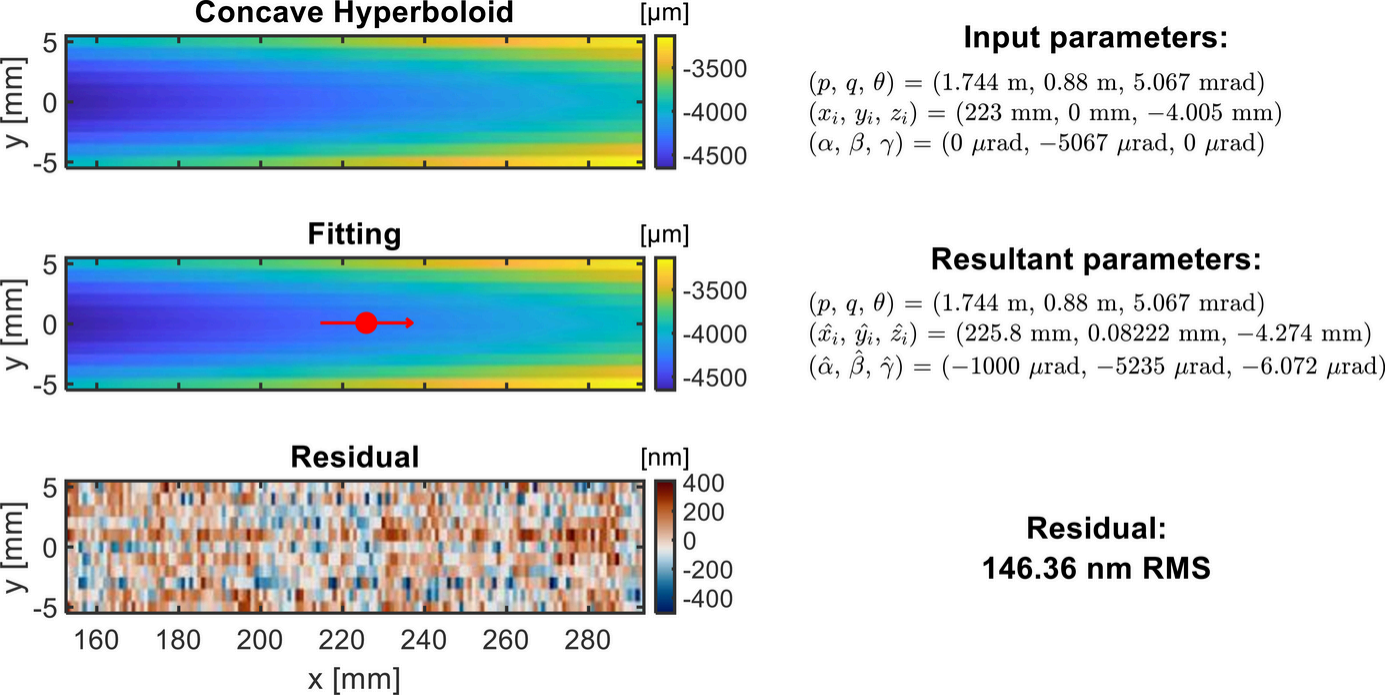
Fitting example with concave hyperboloid height data. The red dot indicates the location of the chief ray intersection from the fitting. The fitting is carried out with fixed *p*, *q*, and θ, and optimized *x*_*i*_, *y*_*i*_, *z*_*i*_, α, β, and γ with boundaries *y*_*i*_ ∈ [−0.5 mm, 0.5 mm] and α ∈ [−1 mrad, 1 mrad].

The fifth example in Fig. 9 ▸ is a 2D height map fitting of a concave hyperboloidal mirror with tolerance to constrain the boundary in the optimization. The chief ray intersection (*x*_*i*_, *y*_*i*_, *z*_*i*_) and rotation angles (α, β, γ) are optimized to fit the measured height surface with the target height. In this example, we set a tolerance [−0.5 mm, 0.5 mm] for *y*_*i*_ and tolerance [−1 mrad, 1 mrad] for α in **p**_*b*_ in equation (9) to constrain the optimization boundaries. In this way, the fitting becomes a constrained optimization problem, and the solution offers a practical approach to making the best use of the mirror. Random noise intrinsic to the metrology data is dominating the residual height map.

We demonstrate several representative real measurement data as examples to validate the effectiveness of the proposed fitting framework. These examples cover a range of common mirror geometries and data types, including 1D slope or height profiles and 2D height maps. The consistency between the fitted and reference shapes illustrates the accuracy and robustness of the method. Overall, the fitting results confirm that the proposed framework is not only theoretically sound but also practically feasible for use in X-ray mirror fabrication and inspection workflows.

## 4. Discussion

In this section, we discuss several aspects of the proposed fitting framework including the practical influences of the fitting parameters and their couplings, as well as the use of Boolean optimization flags and tolerance boundaries. We will also address the use of the existing fitting framework to fit paraboloids, parabolic cylinders, spheres, and circular cylinders, and potential extension for other shapes.

### 4.1. Practical influences of fitting parameters

Understanding the practical meanings and influences of these parameters helps interpret the fitting results and guides necessary and minimal refinements to the mirror surface, such as optical polishing during its fabrication or mechanical adjustments. For example, the misalignment between the metrology coordinate with the optical area in rotation angle γ will introduce a ‘twist-like’ surface figure error. If the surface fitting does not include optimization of γ, the ‘twist’ may be mistakenly interpreted as a surface imperfection.

**Figure 10:**
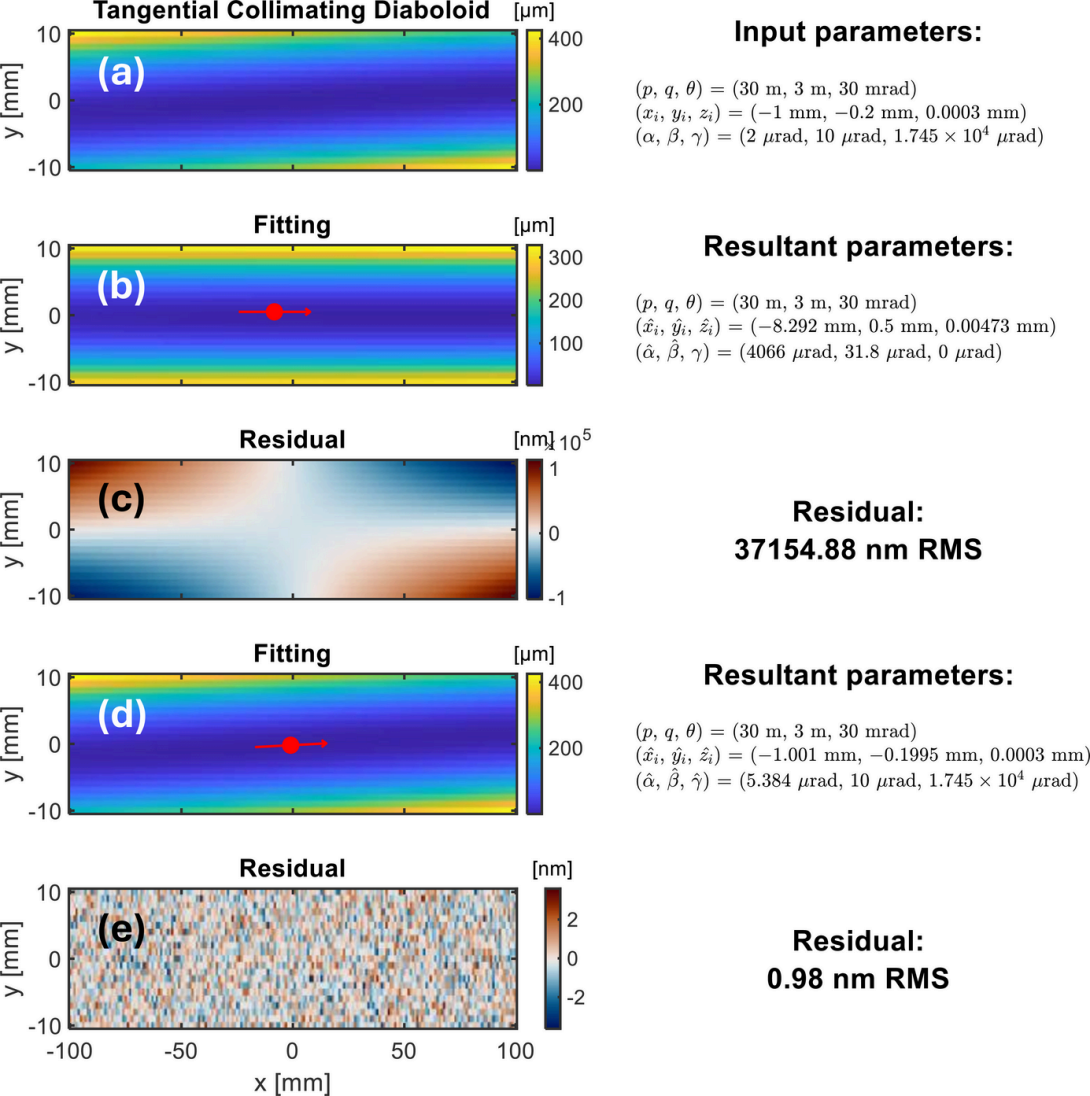
Fitting example with 2D tangential collimating diaboloid height data (*a*). The red dot indicates the location of the chief ray intersection from the fitting. (*b*) The fitting is carried out with fixed *p*, *q*, and θ, and optimized *x*_*i*_, *y*_*i*_, *z*_*i*_, α, and β. (*c*) The residual height of (*b*) is dominated by a twist. (*d*) The fitting is carried out with fixed *p*, *q*, and θ, and optimized *x*_*i*_, *y*_*i*_, *z*_*i*_, α, β, and γ. (*e*) The residual height of (*d*) is random noise only.

The example shown in Fig. 10 ▸ is the 2D height map fitting of a simulated tangential collimating diaboloidal mirror. As shown in Fig. 10 ▸(*a*), the shape parameters are *p* = 30 m, *q* = 3 m (in the sagittal direction), and θ = 30 mrad. The pose parameters are set as (*x*_*i*_, *y*_*i*_, *z*_*i*_) = (−1 mm, −0.2 mm, 0.003 mm) and (α, β, γ) = (2 µrad, 10 µrad, 17.45 mrad). By purpose, we simulate an obvious misalignment in γ between the optical area on the mirror and the metrology system. Normally distributed random noise with σ = 1 nm RMS are added to this simulated surface height map.

In Fig. 10 ▸(*b*), if we only optimize the chief ray intersection (*x*_*i*_, *y*_*i*_, *z*_*i*_) and other rotation angles α and β without including the angle γ in the fitting process, the height residual can be dominated by a large ‘twist’, or say astigmatism at 45°, as shown in Fig. 10 ▸(*c*). In this optimization, the tolerance of *y*_*i*_ is set to be [−0.5 mm, 0.5 mm].

In contrast, the chief ray intersection (*x*_*i*_, *y*_*i*_, *z*_*i*_) and rotation angles (α, β, γ) are optimized as shown in Fig. 10 ▸(*d*). Similarly, the tolerance of *y*_*i*_ is set to be [−0.5 mm, 0.5 mm]. The height residual map in Fig. 10 ▸(*e*) mainly contains random noise only. Insight into the practical influences of these parameters enables a better understanding of the fitting results and avoids unnecessary corrections on the mirror surface.

One thing to highlight is that, even if there is a large misalignment between the effective optical area and the metrology instrument (γ = 17.45 mrad ≃ 1°) in this simulation example, it demonstrates the validity of the proposed surface fitting framework.

### 4.2. Couplings of fitting parameters

Selecting fitting parameters is crucial for accurately modeling the mirror surface shape. Each parameter in the mathematical expressions corresponds to a physical characteristic of the mirror. For example, for elliptic or hyperbolic cylinders, *x*_*i*_ coupled with *p* and *q* values majorly influences curvature of the mirror surface shape. The grazing incidence angle θ affects both curvature and coma terms on the mirror surface shape. When fitting ellipsoids or hyperboloids, some pose parameters like *y*_*i*_, α, and γ can be coupled with each other.

### 4.3. Boolean optimization flags and tolerance boundaries

Since some parameters are coupled, it is important to avoid optimizing them simultaneously when using Boolean optimization flags **p**_*b*_ in equation (9). Different optimization algorithms, such as the trust-region reflective method or the Levenberg–Marquardt algorithm, may yield significantly different parameter combinations to ‘best explain’ the metrology data due to inherent ambiguities.

In such cases, it is often more practical to use tolerance boundaries instead of Boolean flags as **p**_*b*_ in equation (9) if the tolerances of the parameters are known. The optimizer will then search for the best solution within the specified boundaries to minimize the residual, resulting in a constrained solution that is practically meaningful.

### 4.4. Analytic expressions for mirror surface shapes

In Section S1 of the supporting information, we summarize the analytical expressions for several kinds of off-axis mirror surface shapes in two conventions. For practical usages, it is straightforward to use the absolute values of distances |*p*| and |*q*|, along with the convex and concave property of the mirror as the convention described in Section S1.1. For code implementation and maintenance in programming, the convention described in Section S1.2 follows the convention in geometrical optics, considering the signs of the distances *p* and *q* based on the real or virtual property of the object and image. It unifies surface expressions (convex or concave, elliptic or hyperbolic), which simplifies the implementation and maintenance in code. In Section S2, we summarize the expressions for sagittal and tangential collimating diaboloids, which can be directly used in the surface fitting framework.

### 4.5. Fitting paraboloids and parabolic cylinders

The expressions for a paraboloid and parabolic cylinder can be derived easily from the expressions for ellipsoid and elliptic cylinder by considering *p* → ±∞ or *q* → ±∞, which delivers four cases of convex/concave and collimating/focusing mirrors. In practice, we find it is accurate enough to use the fitting function for ellipsoids and elliptic cylinders to deal with the paraboloids and parabolic cylinders fitting problem if the distance of the long-arm is set with an extremely large value (such as 10^30^ m) to approximate infinity.

### 4.6. Fitting spheres and circular cylinders

Spheres can be treated as special ellipsoids, and circular cylinders can be considered as special elliptic cylinders with *p* = *q* = *R* (*R* is the radius of curvature of the sphere or circular cylinder in tangential direction) and θ = π/2. It is obvious that spheres and circular cylinders can be fitted by using the proposed framework based on the ellipsoid and elliptic cylinder fitting, after some modifications.

In theory, there are two ways to modify the framework to fit spheres and circular cylinders. One is by adding constraints to restrict the *p* = *q* in optimization while fixing θ = π/2. The other method is optimizing *R* as a parameter while fixing θ = π/2 instead of using *p* and *q* as two constrained parameters. The first way requires minimum modifications on the existing code, but the results are not as stable as that of the second method. Therefore, we suggest to use the second approach for better performance in practice.

### 4.7. Easy extension to more complex shapes

The framework is designed to be both flexible and extensible. While this work focuses on standard shapes such as elliptic and hyperbolic cylinders, the same structure, especially the middle layers in the framework, can be readily adapted to support more complex mirror surface profiles. By defining additional mathematical expressions in the bottom layer and integrating them into the fitting process, the framework can accommodate a broader range of geometries used in advanced X-ray optics, ensuring its continued applicability for future developments.

### 4.8. Open source

The fitting framework is available as open-source codes (Huang & Xu, 2025 ▸) in MATLAB and Python to promote academic collaboration and support further development. The community can access the source code, contribute improvements, and tailor the framework to their specific needs. By sharing the framework with the community, we aim to facilitate the development of high-quality X-ray mirrors and enhance the performance of X-ray optics in various applications. It would be highly beneficial and would aid standardization and transparency of mirror metrology data analysis for future round-robin metrology exercises between academia and industry.

### 4.9. Limitations

This framework is focusing on surface fitting of the *ex situ* mirror metrology data, so we do not involve the aspect of weighting, which considers the non-uniform distribution of the beam footprint on the mirror surface (Goldberg & Yashchuk, 2016 ▸; Goldberg & La Fleche, 2024 ▸). The weighting capability is interesting in the mirror alignment and the wavefront control with adaptive X-ray mirrors, because it can reduce the impact of the figure errors at edges and corners where the beam intensity is extremely low.

The toroidal mirror is another type of X-ray mirror commonly used at synchrotron beamlines, which is not yet implemented in the current framework.

## 5. Conclusion

We have presented a comprehensive framework for fitting the surface shapes of X-ray mirrors used in SR and FEL facilities. By defining standard shapes using mathematical expressions, generating theoretical surface profiles considering pose parameters, and fitting to the measured data, our framework ensures accurate characterization and optimization of X-ray mirror surfaces. The results demonstrate the effectiveness of our approach in detecting and quantifying surface deviations, thereby enhancing the performance of X-ray optics. The flexibility and extensibility of the framework make it a valuable tool for future developments in X-ray mirror fabrication and characterization. We have also made the framework open source to encourage collaboration and further improvements by the scientific community. The open-source implementation is freely available at https://github.com/nsls2omf/xmf, enabling direct adoption and further improvement by the scientific community.

## 6. Related literature

The following reference, not cited in the main body of the paper, has been cited in the supporting information: Klementiev & Chernikov (2014 ▸).

## Supplementary Material

Supporting Sections S1 and S2. DOI: 10.1107/S1600577525011282/mo5313sup1.pdf

## Figures and Tables

**Table 1:** Standard shape expressions for different X-ray mirrors (equations can be found in the supporting information)

	Expressions with |*p*| and |*q*|			Expressions with *p* and *q*	
Mirror type		Height	Slope	Height	Slope
Elliptic cylinder	Convex	Eq.(S26)	Eq.(S33)	Eq.(S43)	Eq.(S45)
	Concave	Eq.(S27)	Eq.(S34)		

Hyperbolic cylinder	Convex	Eq.(S30)	Eq.(S37)	Eq.(S44)	Eq.(S46)
	Concave	Eq.(S31)	Eq.(S38)		

Ellipsoid	Convex	Eq.(S19)		Eq.(S41)	
	Concave	Eq.(S20)			

Hyperboloid	Convex	Eq.(S23)		Eq.(S42)	
	Concave	Eq.(S24)			

Sagittal collimating diaboloid	Concave	Eq.(S54)			
Tangential collimating diaboloid	Concave	Eq.(S60)			

**Table 2:** Surface generation for different mirror types and dimensions

Mirror type	Dimension	Height	Slope
Elliptic or hyperbolic cylinder	1D	Equation (7)	Equation (8)
	2D	Equation (6)	

Ellipsoid/hyperboloid/diaboloid	2D	Equation (5)	

**Table 3:** Fitting parameters for different mirror shapes

		Parameters to optimize	
Example optics: length *L* × width *W*	Target parameters	Selection A	Selection B
1D concave elliptic cylinder slope	*p* = 65.63 m		θ
*L* = 200 mm	*q* = 0.31 m	*x*_*i*_	*x*_*i*_
(Fig. 5 ▸)	θ = 3 mrad	β	β

1D concave elliptic cylinder height	*p* = 65.63 m		θ
*L* = 200 mm	*q* = 0.31 m	*x*_*i*_, *z*_*i*_	*x*_*i*_, *z*_*i*_
(Fig. 6 ▸)	θ = 3 mrad	β	β

2D concave hyperbolic cylinder height	*p* = 30 m		
*L* = 53 mm, *W* = 2 mm	*q* = 0.3 m	*x*_*i*_, *z*_*i*_	–
(Fig. 7 ▸)	θ = 3 mrad	α, β, γ	

2D concave ellipsoid height	*p* = 40 m		
*L* = 150 mm, *W* = 10 mm	*q* = 1.882 m	*x*_*i*_, *z*_*i*_	–
(Fig. 8 ▸)	θ = 5.427 mrad	α, β, γ	

2D concave hyperboloid height	*p* = 1.76 m	*x*_*i*_, *z*_*i*_, β, γ	
*L* = 140 mm, *W* = 10 mm	*q* = 0.88 m	*y*_*i*_ ∈ [−0.5 mm, 0.5 mm]	–
(Fig. 9 ▸)	θ = 5.1 mrad	α ∈ [−1 mrad, 1 mrad]	

## Data Availability

The data supporting the results reported in the article can be accessed in the repository (Huang & Xu, 2025 ▸).
