# Supplementary material for: Framework for X-ray mirror surface shape fitting
Source: J Synchrotron Radiat. 2026 Jan 22;33(Pt 2):278–88. doi: 10.1107/S1600577525011282 (PMC12948005; doi:10.1107/S1600577525011282)
Supplement: Supplementary file 1 [file s-33-00278-sup1.pdf]

JOURNAL OF SYNCHROTRON RADIATION

Volume 33 (2026)

Supporting information for article:

Framework for X-ray mirror surface shape fitting

Lei Huang, Ruochen Xu, Tianyi Wang, Jumpei Yamada, Joseph Dvorak, Corey Austin, Albert Van Eeckhout, Josep Nicolàs Roman, Kenneth Goldberg and Mourad Idir

S1 Standard shape expressions of ellipsoid, hyperboloid, elliptic cylinder, and hyperbolic cylinder in mirror coordinates

We derive the mathematical expressions of the standard shape of grazing-incidence X-ray mirror surface in the mirror coordinates. To simplify the derivation of symmetrical shape expressions, we assume the beam travels from left to right, with the mirror surface oriented upwards. In the derivation process, we use the absolute values of the object and image distances $|p|$ and $|q|$ at first, and then we will introduce the signs of the object distance p and image distance q to unify the expressions for convex and concave mirrors following the convention of geometrical optics.

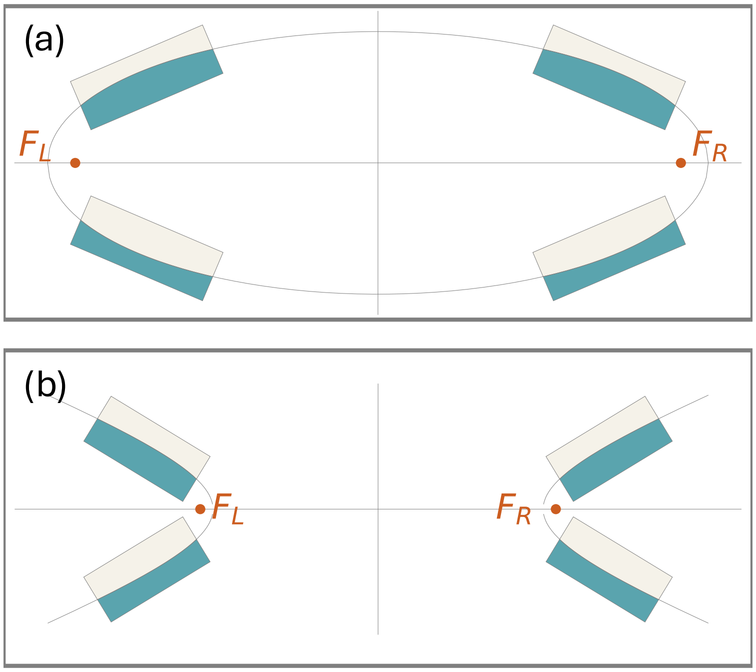

Fig. S1: Based on the central symmetry, we simplify 8 cases to 4 cases for either ellipse (a) or hyperbola (b). Only the mirror sections facing up (4 cases in color of aqua blue) are considered in the following derivations.

Considering possible locations of a mirror surface on the top or bottom, left or right sections, there are 8 cases no matter for ellipses or for hyperbolas as shown in Fig. S1. In the following derivations of this work, we focus on the mirrors facing up (4 cases) in the sketch to simplify the mathematical expression derivation.

S1.1 Standard shape expression with absolute values of object and image distances $|p|$ and $|q|$

In practical beamline applications, the distances are given with positive values even in an optical layout. The widely used KB mirrors or ellipsoidal mirror have positive object distance p and image distance q. Therefore, it becomes a common practice to use the object and image distances in

positive values, so we first use the absolute values of p and q (*i.e.* $|p|$ and $|q|$) to describe the standard shapes of the grazing-incidence X-ray mirror surface. This gives a convenient interface for practical beamline usages.

S1.1.1 Ellipsoid and hyperboloid

We derive the equations for both concave and convex ellipsoids and hyperboloids together to reveal their connections and differences in expression. Once the height expressions of 2D curved ellipsoids and hyperboloids are derived, it is straightforward to derive the height and slope expressions of their 1D tangential curved cylindrical surfaces (elliptic and hyperbolic cylinders).

S1.1.1.1 Implicit equation of an ellipsoid

Based on the definition of the ellipsoid, the sum of distances between an arbitrary point M on the ellipsoid surface and the two foci F_L and F_R is a constant, which is equal to the sum of the absolute values of the object distance $|p|$ and the image distance $|q|$.

$$|F_L M| + |M F_R| = |p| + |q|. \tag{S1}$$

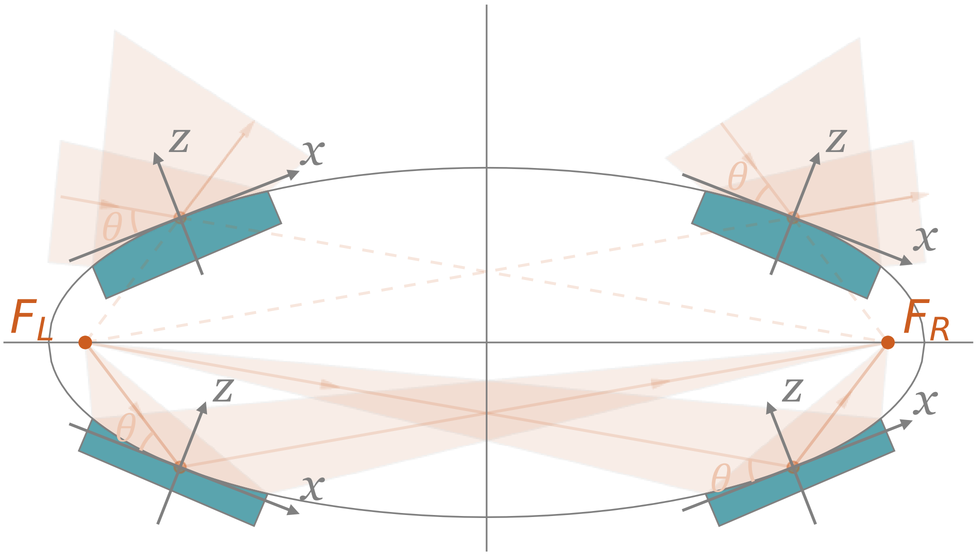

Fig. S2: Sketch of elliptic sections at the top and bottom.

As illustrated in Fig. S2, the convex ellipsoid at the top can be expressed as

$$\sqrt{(x+|q|\cos\theta)^2 + y^2 + (z+|q|\sin\theta)^2} + \sqrt{(x-|p|\cos\theta)^2 + y^2 + (z+|p|\sin\theta)^2} = |p| + |q|. \tag{S2}$$

The concave ellipsoid at the bottom can be expressed as

$$\sqrt{(x+|p|\cos\theta)^2 + y^2 + (z-|p|\sin\theta)^2} + \sqrt{(x-|q|\cos\theta)^2 + y^2 + (z-|q|\sin\theta)^2} = |p| + |q|, \tag{S3}$$

where the angle θ is the grazing angle at the chief ray intersection where the origin of the mirror coordinate system is defined with zero slope and height.

S1.1.1.2 Implicit equation of a hyperboloid

By definition, a hyperboloid is characterized by the absolute difference between the distances from any point M on its surface to the two foci F_L and F_R. This absolute difference is constant and equals the absolute difference between the object distance $|p|$ and the image distance $|q|$.

$$\big||F_LM| - |MF_R|\big| = \big||p| - |q|\big|. \tag{S4}$$

Consider hyperboloid with $|p| > |q|$ and $|p| < |q|$ cases, we have

$$\big||F_LM| - |MF_R|\big| = \begin{cases} |F_LM| - |MF_R| = |p| - |q|, & |p| > |q|, \\ -|F_LM| + |MF_R| = -(|p| - |q|), & |p| < |q|. \end{cases}$$

$$\therefore |F_LM| - |MF_R| = |p| - |q|. \tag{S5}$$

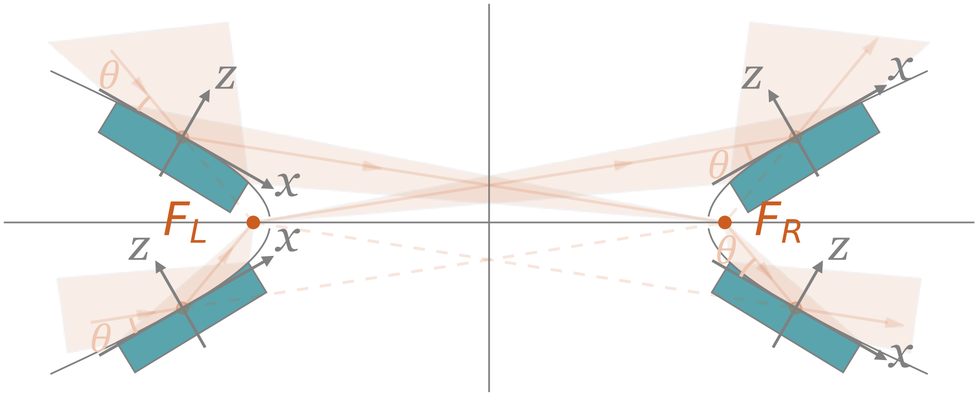

Fig. S3: Sketch of hyperbolic sections on the left and right branches.

As illustrated in Fig. S3, the left hyperboloid can be expressed as

$$\sqrt{(x - |p|\cos\theta)^2 + y^2 + (z + |p|\sin\theta)^2} - \sqrt{(x - |q|\cos\theta)^2 + y^2 + (z - |q|\sin\theta)^2} = |p| - |q|, \tag{S6}$$

and the right hyperboloid can be expressed as

$$\sqrt{(x + |p|\cos\theta)^2 + y^2 + (z - |p|\sin\theta)^2} - \sqrt{(x + |q|\cos\theta)^2 + y^2 + (z + |q|\sin\theta)^2} = |p| - |q|, \tag{S7}$$

where θ is grazing angle at the chief ray intersection where the origin of the mirror coordinate system is defined with zero slope and height.

S1.1.1.3 Unified derivation for ellipsoid and hyperboloid expressions

Combining both ellipsoid in Eq. (S1) and hyperboloid Eq. (S5), we can summarize the distance relations with a unified sign symbol to present the different operations for the top ellipsoid, bottom ellipsoid, left hyperboloid and right hyperboloid as

$$|F_L M| \underset{--}{\overset{+}{+}} |MF_R| = |p| \underset{--}{\overset{+}{+}} |q|, \tag{S8}$$

where the associated position of the unified sign symbol is illustrated in Fig. S4.

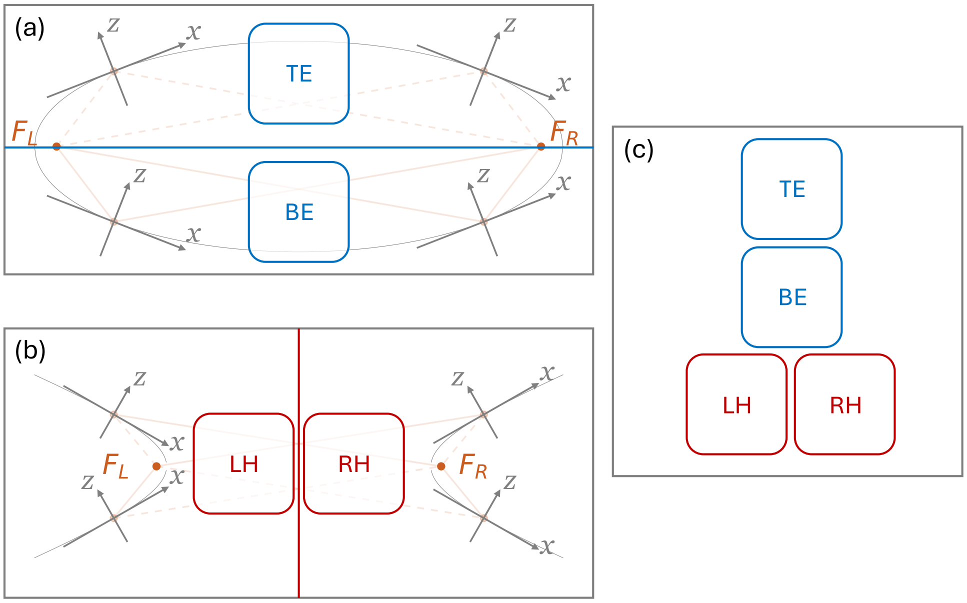

Fig. S4: Sketch of the unified sign symbol from top ellipse (TE) and bottom ellipse (BE) sections (a) and left hyperbola (LH) and right hyperbola (RH) branches (b). (c) The order of the signs in the unified sign symbol.

Combining Eqs. (S2), (S3), (S6), and (S7), implicit equations of the four cases are unified as

$$\sqrt{(x \underset{-+}{\overset{-}{+}} |p|\cos\theta)^2 + y^2 + (z \underset{+-}{\overset{+}{-}} |p|\sin\theta)^2} \underset{--}{\overset{+}{+}} \sqrt{(x \underset{-+}{\overset{+}{-}} |q|\cos\theta)^2 + y^2 + (z \underset{-+}{\overset{+}{-}} |q|\sin\theta)^2} = |p| \underset{--}{\overset{+}{+}} |q|. \tag{S9}$$

By squaring both sides of the equation and rearranging the terms in descending powers of z, we obtain a quadratic equation in z.

$$\begin{aligned} &\left[(|p| \underset{--}{\overset{+}{+}} |q|)^2 - (|p| \underset{++}{\overset{-}{-}} |q|)^2 \sin^2\theta\right] z^2 \\ &+ \left[2x(|p| + |q|)(|p| - |q|)\sin\theta\cos\theta \underset{-+}{\overset{+}{-}} 4|pq|(|p| \underset{--}{\overset{+}{+}} |q|)\sin\theta\right] z \\ &+(|p| \underset{--}{\overset{+}{+}} |q|)^2 (x^2\sin^2\theta + y^2) = 0. \end{aligned} \tag{S10}$$

Describing it in the standard quadratic equation as $Az^2 + Bz + C = 0$, the coefficients are given

as

$$
\begin{aligned}
A &= (|p| \underset{--}{\overset{+}{+}} |q|)^2 - (|p| \underset{++}{\overset{-}{-}} |q|)^2 \sin^2\theta, \\
B(x) &= 2x(|p| + |q|)(|p| - |q|)\sin\theta\cos\theta \underset{-+}{\overset{+}{-}} 4|pq|(|p| \underset{--}{\overset{+}{+}} |q|)\sin\theta, \\
C(x,y) &= (|p| \underset{--}{\overset{+}{+}} |q|)^2 (x^2\sin^2\theta + y^2).
\end{aligned}
\tag{S11}
$$

Solving the quadratic equation with the constraint that the origin of the standard mirror coordinates locates on the mirror surface, we obtain the constrained solutions as follows:

$$
\begin{aligned}
& z(x,y) = \frac{-B \pm \sqrt{B^2 - 4AC}}{2A} = \frac{-B \pm \sqrt{\Delta}}{2A}, \\
& \text{subject to: } z(0,0) = 0.
\end{aligned}
\tag{S12}
$$

We can utilize the constraint $z(0,0) = 0$ to determine the solution for concave and convex mirrors.

$$
\because \quad C(0,0) = 0, \qquad \therefore z(0,0) = \frac{-B(0) \pm \left|B(0)\right|}{2A}.
$$

By analyzing the signs of p, q, and θ, we can classify the convex and concave cases for ellipsoids and hyperboloids.

$$
\because \quad |p| > 0, \quad |q| > 0, \quad \theta > 0,
$$

$$
\therefore \quad B(0) = \begin{cases}
4|pq|(|p| + |q|)\sin\theta > 0, & \text{Top ellipsoid (Convex ellipsoid)} \\
-4|pq|(|p| + |q|)\sin\theta < 0, & \text{Bottom ellipsoid (Concave ellipsoid)} \\
-4|pq|(|p| - |q|)\sin\theta \begin{cases} < 0, & |p| > |q| \quad \text{Left hyperboloid (Concave hyperboloid)} \\ > 0, & |p| < |q| \quad \text{Left hyperboloid (Convex hyperboloid)} \end{cases} \\
4|pq|(|p| - |q|)\sin\theta \begin{cases} > 0, & |p| > |q| \quad \text{Right hyperboloid (Convex hyperboloid)} \\ < 0, & |p| < |q| \quad \text{Right hyperboloid (Concave hyperboloid)} \end{cases}
\end{cases}
$$

$$\therefore \quad B(0) = \begin{cases} > 0, & \text{Convex ellipsoid or convex hyperboloid} \\ < 0, & \text{Concave ellipsoid or concave hyperboloid} \end{cases}$$

$\therefore$ For convex ellipsoid or convex hyperboloid,

$$z(0,0) = \frac{-B(0) \pm B(0)}{2A} = \begin{cases} 0, & \text{using } z = \dfrac{-B + \sqrt{B^2 - 4AC}}{2A} \\ \dfrac{-B(0)}{A} \neq 0, & \text{using } z = \dfrac{-B - \sqrt{B^2 - 4AC}}{2A} \end{cases}$$

For concave ellipsoid or concave hyperboloid,

$$z(0,0) = \frac{-B(0) \mp B(0)}{2A} = \begin{cases} \dfrac{-B(0)}{A} \neq 0, & \text{using } z = \dfrac{-B + \sqrt{B^2 - 4AC}}{2A} \\ 0, & \text{using } z = \dfrac{-B - \sqrt{B^2 - 4AC}}{2A} \end{cases}$$

Therefore, we get the solution that can be determined using the parameters in Eq. (S11).

$$z(x,y) = \begin{cases} \dfrac{-B+\sqrt{B^2 - 4AC}}{2A} = \dfrac{-B+\sqrt{\Delta}}{2A}, & \text{Convex ellipsoid or convex hyperboloid} \\ \dfrac{-B-\sqrt{B^2 - 4AC}}{2A} = \dfrac{-B-\sqrt{\Delta}}{2A}, & \text{Concave ellipsoid or concave hyperboloid} \end{cases} \tag{S13}$$

The discriminant $\Delta = B^2 - 4AC$ can be simplified as

$$\begin{aligned} \Delta &= B^2 - 4AC \\ &= 4(|p| \underset{--}{\overset{+}{+}} |q|)^2 \sin^2\theta \left\{ \underset{++}{\overset{-}{-}} 4|pq|x^2 \underset{-+}{\overset{+}{-}} 4|pq|(|p| \underset{++}{\overset{-}{-}} |q|) x\cos\theta + 4p^2q^2 - \left[\frac{(|p| \underset{--}{\overset{+}{+}} |q|)^2}{\sin^2\theta} - (|p| \underset{++}{\overset{-}{-}} |q|)^2 \right] y^2 \right\} \end{aligned} \tag{S14}$$

Considering the concave and convex cases for left and right hyperboloid, we have

$$\underset{-}{+}\sqrt{\Delta} = \begin{cases} \underset{-+}{\overset{+}{-}} \sqrt{\Delta}, & |p| > |q| \\ \underset{+-}{\overset{+}{-}} \sqrt{\Delta}, & |p| < |q| \end{cases} \tag{S15}$$

$$\begin{aligned} \underset{-}{+}\sqrt{\Delta} &= \underset{-}{+} 2\sin\theta \left| |p| \underset{--}{\overset{+}{+}} |q| \right| \sqrt{ \underset{++}{\overset{-}{-}} 4|pq|x^2 \underset{-+}{\overset{+}{-}} 4|pq|(|p| \underset{++}{\overset{-}{-}} |q|) x\cos\theta + 4p^2q^2 - \left[\frac{(|p| \underset{--}{\overset{+}{+}} |q|)^2}{\sin^2\theta} - (|p| \underset{++}{\overset{-}{-}} |q|)^2 \right] y^2 } \\ &= \underset{-+}{\overset{+}{-}} 2(|p| \underset{--}{\overset{+}{+}} |q|) \sin\theta \sqrt{ \underset{++}{\overset{-}{-}} 4|pq|x^2 \underset{-+}{\overset{+}{-}} 4|pq|(|p| \underset{++}{\overset{-}{-}} |q|) x\cos\theta + 4p^2q^2 - \left[(|p| \underset{--}{\overset{+}{+}} |q|)^2 - (|p| \underset{++}{\overset{-}{-}} |q|)^2 \sin^2\theta \right] \frac{y^2}{\sin^2\theta} } \end{aligned} \tag{S16}$$

Finally, we can expand the solution expression in Eq. (S13) as

$$z(x,y) = \frac{-B \pm \sqrt{\Delta}}{2A} = \frac{-\left[2x(|p|+|q|)(|p|-|q|)\sin\theta\cos\theta \underset{-+}{\overset{+}{-}} 4|pq|(|p| \underset{--}{\overset{+}{+}} |q|)\sin\theta\right] \pm \sqrt{\Delta}}{2\left[(|p| \underset{--}{\overset{+}{+}} |q|)^2 - (|p| \underset{++}{\overset{-}{-}} |q|)^2 \sin^2\theta\right]} \tag{S17}$$

$$z(x,y) = \frac{(|p| \underset{--}{\overset{+}{+}} |q|)\sin\theta \left[-x(|p| \underset{++}{\overset{-}{-}} |q|)\cos\theta \underset{+-}{\overset{-}{+}} 2|pq| \underset{-+}{\overset{+}{-}} \sqrt{\underset{++}{\overset{-}{-}} 4|pq|x^2 \underset{-+}{\overset{+}{-}} 4|pq|(|p| \underset{++}{\overset{-}{-}} |q|)x\cos\theta + 4p^2q^2 - \left[(|p| \underset{--}{\overset{+}{+}} |q|)^2 - (|p| \underset{++}{\overset{-}{-}} |q|)^2\sin^2\theta\right]\frac{y^2}{\sin^2\theta}}\right]}{(|p| \underset{--}{\overset{+}{+}} |q|)^2 - (|p| \underset{++}{\overset{-}{-}} |q|)^2\sin^2\theta} \tag{S18}$$

From this unified expression in Eq. (S18), one can easily derive the explicit expressions for ellipsoids and hyperboloids.

S1.1.1.4 Explicit expressions of an ellipsoid and a hyperboloid

Based on Eq. (S18), the explicit expressions of ellipsoid and hyperboloid in each case are listed below.

The explicit expression of a convex ellipsoid is

$$z(x,y) = \frac{(|p|+|q|)\sin\theta\left[-x(|p|-|q|)\cos\theta - 2|pq| + \sqrt{-4|pq|x^2 + 4|pq|(|p|-|q|)x\cos\theta + 4p^2q^2 - \left[(|p|+|q|)^2 - (|p|-|q|)^2\sin^2\theta\right]\frac{y^2}{\sin^2\theta}}\right]}{(|p|+|q|)^2 - (|p|-|q|)^2\sin^2\theta} \tag{S19}$$

The explicit expression of a concave ellipsoid is

$$z(x,y) = \frac{(|p|+|q|)\sin\theta\left[-x(|p|-|q|)\cos\theta + 2|pq| - \sqrt{-4|pq|x^2 - 4|pq|(|p|-|q|)x\cos\theta + 4p^2q^2 - \left[(|p|+|q|)^2 - (|p|-|q|)^2\sin^2\theta\right]\frac{y^2}{\sin^2\theta}}\right]}{(|p|+|q|)^2 - (|p|-|q|)^2\sin^2\theta} \tag{S20}$$

The explicit expression of a Left Hyperboloid (LH) is

$$z_{LH}(x,y) = \frac{(|p|-|q|)\sin\theta\left[-x(|p|+|q|)\cos\theta + 2|pq| - \sqrt{4|pq|x^2 - 4|pq|(|p|+|q|)x\cos\theta + 4p^2q^2 - \left[(|p|-|q|)^2 - (|p|+|q|)^2\sin^2\theta\right]\frac{y^2}{\sin^2\theta}}\right]}{(|p|-|q|)^2 - (|p|+|q|)^2\sin^2\theta} \tag{S21}$$

The explicit expression of a Right Hyperboloid (RH) is

$$z_{RH}(x,y) = \frac{(|p|-|q|)\sin\theta\left[-x(|p|+|q|)\cos\theta - 2|pq| + \sqrt{4|pq|x^2 + 4|pq|(|p|+|q|)x\cos\theta + 4p^2q^2 - \left[(|p|-|q|)^2 - (|p|+|q|)^2\sin^2\theta\right]\frac{y^2}{\sin^2\theta}}\right]}{(|p|-|q|)^2 - (|p|+|q|)^2\sin^2\theta} \tag{S22}$$

The explicit expression of convex hyperboloid is

$$z(x,y) = \begin{cases} z_{RH}(x,y), & |p| > |q| \\ z_{LH}(x,y), & |p| < |q| \end{cases} \tag{S23}$$

The explicit expression of concave hyperboloid is

$$z(x,y) = \begin{cases} z_{LH}(x,y), & |p| > |q| \\ z_{RH}(x,y), & |p| < |q| \end{cases} \tag{S24}$$

S1.1.2 Elliptic cylinder and hyperbolic cylinder

S1.1.2.1 Height expressions of an elliptic cylinder and a hyperbolic cylinder

For a tangential curved case, we can have the height expression by sectioning on $y = 0$ plane. The explicit expression of surface height is

$$z(x) = \frac{(|p| \underset{--}{\overset{+}{+}} |q|)\sin\theta \left[-x(|p| \underset{++}{\overset{-}{-}} |q|)\cos\theta \underset{+-}{\overset{-}{+}} 2|pq| \underset{-+}{\overset{+}{-}} 2\sqrt{\underset{++}{\overset{-}{-}} |pq|x^2 \underset{-+}{\overset{+}{-}} |pq|(|p| \underset{++}{\overset{-}{-}} |q|)x\cos\theta + p^2q^2} \right]}{(|p| \underset{--}{\overset{+}{+}} |q|)^2 - (|p| \underset{++}{\overset{-}{-}} |q|)^2 \sin^2\theta} \tag{S25}$$

Therefore, the explicit expression of a convex elliptic cylinder surface height is

$$z(x) = \frac{(|p| + |q|)\sin\theta \left[-x(|p| - |q|)\cos\theta - 2|pq| + 2\sqrt{-|pq|x^2 + |pq|(|p| - |q|)x\cos\theta + p^2q^2} \right]}{(|p| + |q|)^2 - (|p| - |q|)^2 \sin^2\theta} \tag{S26}$$

The explicit expression of a concave elliptic cylinder surface height is

$$z(x) = \frac{(|p| + |q|)\sin\theta \left[-x(|p| - |q|)\cos\theta + 2|pq| - 2\sqrt{-|pq|x^2 - |pq|(|p| - |q|)x\cos\theta + p^2q^2} \right]}{(|p| + |q|)^2 - (|p| - |q|)^2 \sin^2\theta} \tag{S27}$$

The explicit expression of a Left Hyperbolic Cylinder (LHC) surface height is

$$z_{LHC}(x) = \frac{(|p| - |q|)\sin\theta \left[-x(|p| + |q|)\cos\theta + 2|pq| - 2\sqrt{|pq|x^2 - |pq|(|p| + |q|)x\cos\theta + p^2q^2} \right]}{(|p| - |q|)^2 - (|p| + |q|)^2 \sin^2\theta} \tag{S28}$$

The explicit expression of a Right Hyperbolic Cylinder (RHC) surface height is

$$z_{RHC}(x) = \frac{(|p| - |q|)\sin\theta \left[-x(|p| + |q|)\cos\theta - 2|pq| + 2\sqrt{|pq|x^2 + |pq|(|p| + |q|)x\cos\theta + p^2q^2} \right]}{(|p| - |q|)^2 - (|p| + |q|)^2 \sin^2\theta} \tag{S29}$$

The explicit expression of a convex hyperbolic cylinder surface height is

$$z(x,y) = \begin{cases} z_{RHC}(x), & |p| > |q| \\ z_{LHC}(x), & |p| < |q| \end{cases} \tag{S30}$$

The explicit expression of a concave hyperbolic cylinder surface height is

$$z(x,y) = \begin{cases} z_{LHC}(x), & |p| > |q| \\ z_{RHC}(x), & |p| < |q| \end{cases} \tag{S31}$$

S1.1.2.2 Tangential slope expressions of an elliptic cylinder and a hyperbolic cylinder

The explicit expression of the tangential slope $\frac{dz(x)}{dx}$ can be derived by taking the first derivative of Eq. (S25).

$$\frac{dz(x)}{dx} = \frac{(|p| \overset{+}{\underset{--}{+}} |q|)\sin\theta}{(|p| \overset{+}{\underset{--}{+}} |q|)^2 - (|p| \overset{-}{\underset{++}{-}} |q|)^2 \sin^2\theta} \left[-(|p| \overset{-}{\underset{++}{-}} |q|)\cos\theta \overset{+}{\underset{-+}{-}} \frac{\overset{-}{\underset{++}{-}} 2|pq|x \overset{+}{\underset{-+}{-}} |pq|(|p| \overset{-}{\underset{++}{-}} |q|)\cos\theta}{\sqrt{\overset{-}{\underset{++}{-}} |pq|x^2 \overset{+}{\underset{-+}{-}} |pq|(|p| \overset{-}{\underset{++}{-}} |q|)x\cos\theta + p^2q^2}} \right] \tag{S32}$$

Therefore, the explicit expression of the tangential slope of a convex elliptic cylinder is

$$\frac{dz(x)}{dx} = \frac{(|p| + |q|)\sin\theta}{(|p| + |q|)^2 - (|p| - |q|)^2 \sin^2\theta} \left[-(|p| - |q|)\cos\theta + \frac{-2|pq|x + |pq|(|p| - |q|)\cos\theta}{\sqrt{-|pq|x^2 + |pq|(|p| - |q|)x\cos\theta + p^2q^2}} \right] \tag{S33}$$

The explicit expression of the tangential slope of a concave elliptic cylinder is

$$\frac{dz(x)}{dx} = \frac{(|p| + |q|)\sin\theta}{(|p| + |q|)^2 - (|p| - |q|)^2 \sin^2\theta} \left[-(|p| - |q|)\cos\theta + \frac{2|pq|x + |pq|(|p| - |q|)\cos\theta}{\sqrt{-|pq|x^2 - |pq|(|p| - |q|)x\cos\theta + p^2q^2}} \right] \tag{S34}$$

The explicit expression of the tangential slope of a left hyperbolic cylinder is

$$\frac{dz_{LHC}(x)}{dx} = \frac{(|p| - |q|)\sin\theta}{(|p| - |q|)^2 - (|p| + |q|)^2 \sin^2\theta} \left[-(|p| + |q|)\cos\theta - \frac{2|pq|x - |pq|(|p| + |q|)\cos\theta}{\sqrt{|pq|x^2 - |pq|(|p| + |q|)x\cos\theta + p^2q^2}} \right] \tag{S35}$$

The explicit expression of the tangential slope of a right hyperbolic cylinder is

$$\frac{dz_{RHC}(x)}{dx} = \frac{(|p| - |q|)\sin\theta}{(|p| - |q|)^2 - (|p| + |q|)^2 \sin^2\theta} \left[-(|p| + |q|)\cos\theta + \frac{2|pq|x + |pq|(|p| + |q|)\cos\theta}{\sqrt{|pq|x^2 + |pq|(|p| + |q|)x\cos\theta + p^2q^2}} \right] \tag{S36}$$

The explicit expression of the tangential slope of a convex hyperbolic cylinder is

$$\frac{dz(x)}{dx} = \begin{cases} \dfrac{dz_{RHC}(x)}{dx}, & |p| > |q| \\ \dfrac{dz_{LHC}(x)}{dx}, & |p| < |q| \end{cases} \tag{S37}$$

The explicit expression of the tangential slope of a concave hyperbolic cylinder is

$$\frac{dz(x)}{dx} = \begin{cases} \dfrac{dz_{LHC}(x)}{dx}, & |p| > |q| \\ \dfrac{dz_{RHC}(x)}{dx}, & |p| < |q| \end{cases} \tag{S38}$$

It is convenient to use these expressions with the absolute values of distance $|p|$ and $|q|$ in beamline applications. However, the signs of p and q are also important to determine the real and virtual object and image. Therefore, we can also use the signs of p and q to determine the concave and convex property of the mirror.

The expressions of the standard shapes of the elliptic cylinder, the ellipsoid, the hyperbolic cylinder, and the hyperboloid have been validated through ray tracing using XRT (Klementiev & Chernikov, 2014).

S1.2 Standard shape expression considering signs of p and q

Following the convention in optics, the sign of the object distance and image distance is determined by whether the object and image are real or virtual. A positive distance indicates a real object or image, while a negative distance corresponds to a virtual object or image.

- For the convex ellipsoid, the object and image are both virtual, so $p < 0$ and $q < 0$.
- For the concave ellipsoid, the object and image are both real, so $p > 0$ and $q > 0$.
- For the left hyperboloid, the object is virtual and the image is real, so $p < 0$ and $q > 0$.
- For the right hyperboloid, the object is real and the image is virtual, so $p > 0$ and $q < 0$.

This convention provides clarity by distinguishing the physical properties of the mirror (such as its concave or convex nature) from the optical properties of the system (such as whether the object and image are real or virtual). Here, we will show that when the signs of p and q are considered the shape expressions become unified and simpler.

S1.2.1 Solution of quadratic equation considering signs of p and q

Following this convention, the coefficients of the quadratic equation shown in Eq. (S11) become unified expressions, which is the beauty of considering the signs of p and q.

$$\begin{aligned} A &= (p+q)^2 - (p-q)^2 \sin^2\theta, \\ B(x) &= 2x(p+q)(p-q)\sin\theta\cos\theta - 4pq(p+q)\sin\theta, \\ C(x,y) &= (p+q)^2(x^2\sin^2\theta + y^2). \end{aligned} \tag{S39}$$

The solution selection follows the same rule in Eq. (S13) based on the convex and concave shape

of the mirror.

$$z(x,y) = \begin{cases} \dfrac{-B+\sqrt{B^2-4AC}}{2A}, & \text{Convex ellipsoid or convex hyperboloid} \\ \dfrac{-B-\sqrt{B^2-4AC}}{2A}, & \text{Concave ellipsoid or concave hyperboloid} \end{cases} \quad \text{(S40)}$$

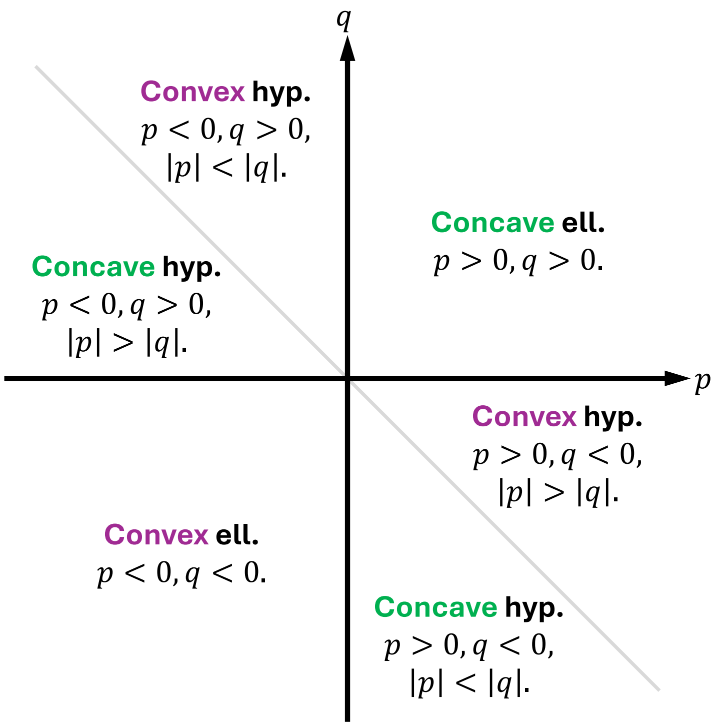

Fig. S5: The classification of the mirrors based on the signs of p and q.

As illustrated in Fig. S5, the convex and concave property is easy to identify by the signs of p and q for ellipsoids.

- If $p < 0$ and $q < 0$, the ellipsoid is convex.
- If $p > 0$ and $q > 0$, the ellipsoid is concave.

For hyperboloids, the signs of p and q are not enough to determine the concave and convex property. We need to compare the absolute values $|p|$ and $|q|$ in addition.

- If $p < 0$, $q > 0$, and $|p| < |q|$, the left hyperboloid is convex.
- If $p < 0$, $q > 0$, and $|p| > |q|$, the left hyperboloid is concave.
- If $p > 0$, $q < 0$, and $|p| < |q|$, the right hyperboloid is concave.
- If $p > 0$, $q < 0$, and $|p| > |q|$, the right hyperboloid is convex.

S1.2.2 Explicit expressions considering signs of p and q

The explicit expressions in Eqs. (S18), (S25), and (S32) are derived from the quadratic equation in Eq. (S11) with considering the convex and concave solutions already. Therefore, with considering the signs of p and q, the explicit expressions can be simplified into the unified form for concave and convex surfaces.

S1.2.2.1 Explicit expressions of an ellipsoid and a hyperboloid

The explicit expressions for an ellipsoid and a hyperboloid in Eq. (S18) can be simplified into two cases: ellipsoid ($pq > 0$) and hyperboloid ($pq < 0$).

For an ellipsoid ($pq > 0$), the explicit height expression is

$$z(x,y) = \frac{(p+q)\sin\theta\left[-x(p-q)\cos\theta + 2pq - \sqrt{-4pqx^2 - 4pq(p-q)x\cos\theta + 4p^2q^2 - \left[(p+q)^2 - (p-q)^2\sin^2\theta\right]\frac{y^2}{\sin^2\theta}}\right]}{(p+q)^2 - (p-q)^2\sin^2\theta} \tag{S41}$$

For a hyperboloid ($pq < 0$), the explicit height expression is

$$z(x,y) = \frac{(p+q)\sin\theta\left[-x(p-q)\cos\theta + 2pq + \sqrt{-4pqx^2 - 4pq(p-q)x\cos\theta + 4p^2q^2 - \left[(p+q)^2 - (p-q)^2\sin^2\theta\right]\frac{y^2}{\sin^2\theta}}\right]}{(p+q)^2 - (p-q)^2\sin^2\theta} \tag{S42}$$

S1.2.2.2 Explicit expressions of elliptic and hyperbolic cylinders

The same operation is applicable to height expression Eq. (S25) and slope expression Eq. (S32) for cylinders.

For an elliptic cylinder ($pq > 0$), the explicit height expression is

$$z(x) = \frac{(p+q)\sin\theta\left[-x(p-q)\cos\theta + 2pq - 2\sqrt{-pqx^2 - pq(p-q)x\cos\theta + p^2q^2}\right]}{(p+q)^2 - (p-q)^2\sin^2\theta} \tag{S43}$$

For a hyperbolic cylinder ($pq < 0$), the explicit height expression is

$$z(x) = \frac{(p+q)\sin\theta\left[-x(p-q)\cos\theta + 2pq + 2\sqrt{-pqx^2 - pq(p-q)x\cos\theta + p^2q^2}\right]}{(p+q)^2 - (p-q)^2\sin^2\theta} \tag{S44}$$

The tangential slope expression of an elliptic cylinder ($pq > 0$) is

$$\frac{dz(x)}{dx} = \frac{(p+q)\sin\theta}{(p+q)^2 - (p-q)^2\sin^2\theta}\left[-(p-q)\cos\theta + \frac{4pqx + 2pq(p-q)\cos\theta}{\sqrt{-pqx^2 - pq(p-q)x\cos\theta + p^2q^2}}\right] \tag{S45}$$

The tangential slope expression of a hyperbolic cylinder ($pq < 0$) is

$$\frac{dz(x)}{dx} = \frac{(p+q)\sin\theta}{(p+q)^2 - (p-q)^2\sin^2\theta}\left[-(p-q)\cos\theta - \frac{4pqx + 2pq(p-q)\cos\theta}{\sqrt{-pqx^2 - pq(p-q)x\cos\theta + p^2q^2}}\right] \tag{S46}$$

These explicit expressions in Eqs. (S41), (S42), (S43), (S44), (S45), and (S46) unify the convex and concave solutions. The implementation of standard mirror shape functions using these explicit expressions makes the maintenance in software coding simpler.

S2 Standard shape expressions of diaboloidal mirror in mirror coordinates

A diaboloidal mirror can focus a beam from a point source P into a focusing line $\mathbf{q}$ in either tangential direction or sagittal direction as shown in Fig. S6, and vice versa. Depending on the direction of the focusing line, we can classify diaboloidal mirrors into sagittal collimating diaboloid (focusing in the tangential direction) and tangential collimating diaboloid (focusing in the sagittal direction). In this case, we can find $p > 0$ and $q > 0$ in the focusing direction, so the expressions with $(|p|, |q|)$ and (p, q) are the same. For simplicity, we use p and q for the rest of equation derivation.

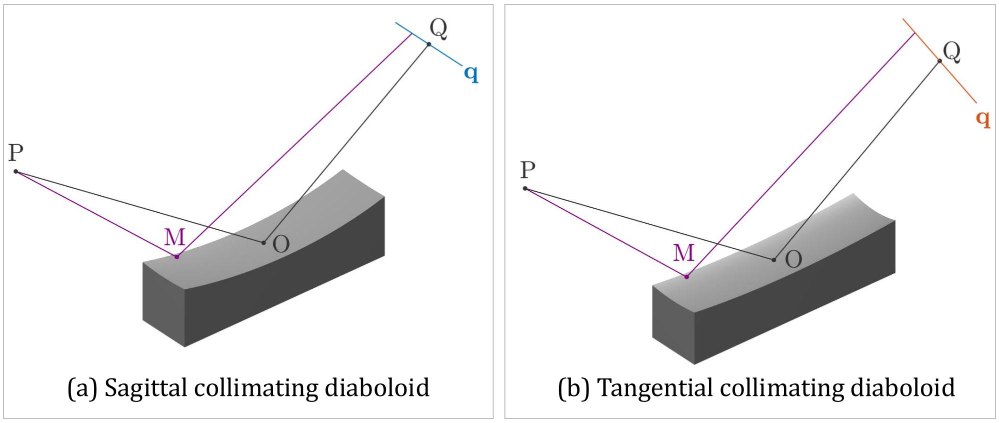

Fig. S6: The diaboloidal mirror can focus from a point source into a line segment, and vice versa. (a) sagittal collimating diaboloid focuses in tangential direction and (b) tangential collimating diaboloid focuses the beam in sagittal direction.

Considering that the chief ray focusing point $Q(x_q, y_q, z_q) = (q\cos\theta, 0, q\sin\theta)$ is on the focusing line $\mathbf{q}$, and the normalized direction vector of the focusing line is

$$\mathbf{v} = (v_x, v_y, v_z) \quad \text{and} \quad \|\mathbf{v}\| = 1, \tag{S47}$$

we can calculate the point-to-line distance between an arbitrary point $M(x, y, z)$ on the mirror

surface and the focusing line $\mathbf{q} = Q + r\mathbf{v}, r \in \mathbb{R}$ by

$$\|M\mathbf{q}\| = \sqrt{[(y-y_q)v_z-(z-z_q)v_y]^2+[(x-x_q)v_z-(z-z_q)v_x]^2+[(x-x_q)v_y-(y-y_q)v_x]^2} \tag{S48}$$

To focus the point source P to line $\mathbf{q}$, the optical path of each beam reflected on the diaboloidal mirror surface should be equal, and we can easily calculate that the optical path of the chief ray is $p+q$, so we have

$$\|PM\| + \|M\mathbf{q}\| = p + q \tag{S49}$$

$$\begin{aligned}&\sqrt{(x+p\cos\theta)^2+y^2+(z-p\sin\theta)^2}\\&+\sqrt{[(y-y_q)v_z-(z-z_q)v_y]^2+[(x-x_q)v_z-(z-z_q)v_x]^2+[(x-x_q)v_y-(y-y_q)v_x]^2}\\&=p+q\end{aligned} \tag{S50}$$

For different orientations of the focusing line vector $\mathbf{v}$, the resulting equation varies in the order of z, and therefore requires different solution methods.

S2.1 Sagittal collimating diaboloid

For a sagittal collimating diaboloid (Dvorak *et al.*, 2025), as shown in Fig. S7, the focusing happens in tangential direction (denoting q as q_t). We symbol the chief ray focus as $Q_t = (x_q, y_q, z_q) = (q_t\cos\theta, 0, q_t\sin\theta)$ and the vector of the focusing line $\mathbf{q}$ is

$$\mathbf{v_t} = (v_x, v_y, v_z) = (0, 1, 0). \tag{S51}$$

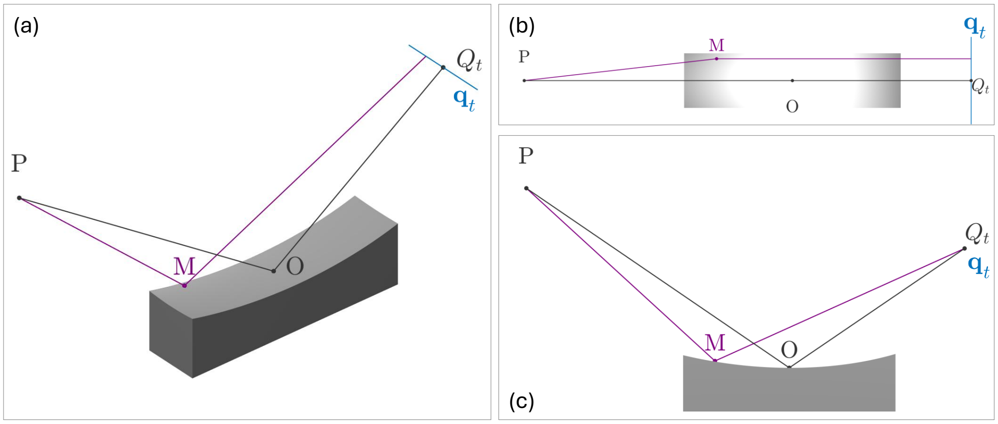

Fig. S7: The sagittal collimating diaboloid focuses in tangential direction in (a) the 3D view, (b) the top view, and (c) the side view.

Eq. (S50) then becomes

$$\sqrt{(x+p\cos\theta)^2+y^2+(z-p\sin\theta)^2}+\sqrt{(x-q_t\cos\theta)^2+(z-q_t\sin\theta)^2} = p+q_t. \tag{S52}$$

It can be derived into a simple quadratic equation $Az^2 + Bz + C = 0$ with coefficients as

$$\begin{aligned} A &= (p - q_t)^2 \cos^2\theta + 4pq_t, \\ B(x,y) &= (p - q_t)y^2 \sin\theta + (p^2 - q_t^2)x \sin 2\theta - 4(p + q_t)pq_t \sin\theta, \\ C(x,y) &= (p + q_t)^2 x^2 \sin^2\theta - (p + q_t)(x\cos\theta - q_t)y^2 - 0.25y^4, \end{aligned} \tag{S53}$$

Since we are only considering concave surface shape for the diaboloidal mirror, the solution for a sagittal collimating diaboloid is

$$z = \frac{-B - \sqrt{B^2 - 4AC}}{2A}, \quad \text{satisfying } z|_{(x,y)=(0,0)} = 0 \tag{S54}$$

S2.2 Tangential collimating diaboloid

For a tangential collimating diaboloid (Yashchuk *et al.*, 2021), the focusing is in sagittal direction (denoting q as q_s). We symbolize the chief ray focus as $Q_s = (x_q, y_q, z_q) = (q_s\cos\theta, 0, q_s\sin\theta)$ and the vector of the focusing line $\mathbf{q}$ is

$$\mathbf{v_s} = (v_x, v_y, v_z) = (-\sin\theta, 0, \cos\theta). \tag{S55}$$

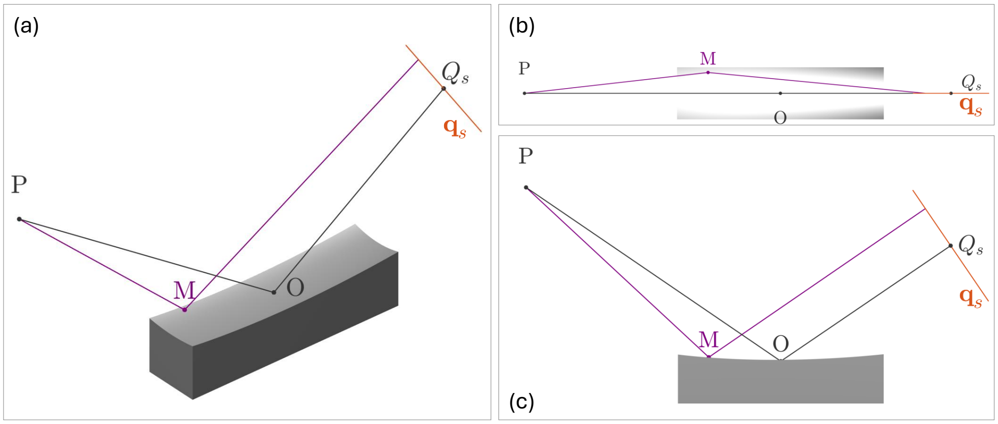

Fig. S8: The tangential collimating diaboloid focuses in sagittal direction in (a) the 3D view, (b) the top view, and (c) the side view.

Eq. (S50) then becomes

$$\sqrt{(x + p\cos\theta)^2 + y^2 + (z - p\sin\theta)^2} + \sqrt{y^2\cos^2\theta + [(x - q_s\cos\theta)\cos\theta + (z - q_s\sin\theta)\sin\theta]^2 + y^2\sin^2\theta} = p + q_s \tag{S56}$$

By simplifying and shifting the term, we have

$$\sqrt{x^2 + y^2 + z^2 + p^2 + 2xp\cos\theta - 2zp\sin\theta} = (p + q_s) - \sqrt{(x\cos\theta + z\sin\theta - q_s)^2 + y^2} \tag{S57}$$

By squaring it, we get

$$
\begin{aligned}
&z^2\cos^2\theta - 2\left[(p-q_s)\sin\theta + x\sin\theta\cos\theta\right]z + x^2\sin^2\theta + 2x(p+q_s)\cos\theta - 2q_s(p+q_s) \\
&= -2(p+q_s)\sqrt{(x\cos\theta + z\sin\theta - q_s)^2 + y^2}
\end{aligned}
\tag{S58}
$$

Eq. (S58) is the same as Eq. (13) in Yashchuk *et al.*, 2021 (Note that the definitions of x and y differ), which is derived by rotating the surface expression in different coordinate systems. Following the reference work (Yashchuk *et al.*, 2021), it turns to a quartic equation $Az^4+Bz^3+Cz^2+Dz+E=0$ with the following coefficients

$$
\begin{aligned}
A &= -\cos^4\theta, \\
B(x) &= 4x\sin\theta\cos^3\theta + 4(p-q_s)\sin\theta\cos^2\theta, \\
C(x) &= -6x^2\sin^2\theta\cos^2\theta + 2x\left[-3p+q_s+(p-3q_s)\cos 2\theta\right]\cos\theta + 4q_s\left[4p\sin^2\theta + (p+q_s)\cos^2\theta\right], \\
D(x) &= 4x^3\sin^3\theta\cos\theta + 2x^2\left[3p+q_s+(p+3q_s)\cos 2\theta\right]\sin\theta + 4x(p+q_s)(2p-q_s)\sin 2\theta - 16pq_s(p+q_s)\sin\theta, \\
E(x,y) &= -x^4\sin^4\theta - 4x^3(p+q_s)\sin^2\theta\cos\theta + 4x^2q_s(p+q_s)\sin^2\theta + 4y^2(p+q_s)^2.
\end{aligned}
\tag{S59}
$$

The solution satisfying $z|_{(x,y)=(0,0)} = 0$ is

$$
z = -\frac{b}{4} - S + \frac{1}{2}\sqrt{-4S^2 - 2k + \frac{m}{S}}, \tag{S60}
$$

where

$$
k = \frac{8c - 3b^2}{8}, \tag{S61}
$$

$$
m = \frac{b^3 - 4bc + 8d}{8}, \tag{S62}
$$

$$
S = \begin{cases}
\frac{1}{2}\sqrt{\frac{1}{3}\left(Q + \frac{\Delta_0}{Q}\right) - \frac{2}{3}k} & \text{when } \Delta_1^2 - 4\Delta_0^3 \geq 0, \\
\frac{1}{2}\sqrt{\frac{2}{3}\sqrt{\Delta_0}\cos\frac{\varphi}{3} - \frac{2}{3}k} & \text{when } \Delta_1^2 - 4\Delta_0^3 < 0,
\end{cases} \tag{S63}
$$

where

$$
Q = \left(\frac{\Delta_1 + \sqrt{\Delta_1^2 - 4\Delta_0^3}}{2}\right)^{1/3}, \tag{S64}
$$

$$
\varphi = \arccos\frac{\Delta_1}{2\sqrt{\Delta_0^3}}, \tag{S65}
$$

$$
\begin{aligned}
\Delta_0 &= c^2 - 3bd + 12e, \\
\Delta_1 &= 2c^3 - 9bcd + 27b^2e + 27d^2 - 72ce,
\end{aligned}
\tag{S66}
$$

with

$$
\begin{aligned}
b &= B/A, \\
c &= C/A, \\
d &= D/A, \\
e &= E/A,
\end{aligned} \tag{S67}
$$

since $A = -\cos^4\theta \neq 0$, when $0 < \theta \ll \frac{\pi}{2}$.

The expressions of the standard shapes of the sagittal collimating diaboloid and the tangential collimating diaboloid have been validated through ray tracing using XRT (Klementiev & Chernikov, 2014).

References

Dvorak, J., Huang, L. & Idir, M. (2025). *Sagittal collimating diaboloid: A new surface figure for higher throughput rixs spectrometers.* In preparation.

Klementiev, K. & Chernikov, R. (2014). *Proc. SPIE*, **9209**, 92090A.

Yashchuk, V. V., Goldberg, K. A., Lacey, I., McKinney, W. R., Sanchez del Rio, M. & Padmore, H. A. (2021). *J. Synchrotron Rad.* **28**, 1031-1040.
